# Dysregulation of miRNAs-COUP-TFII-FOXM1-CENPF axis contributes to the metastasis of prostate cancer

**DOI:** 10.1038/ncomms11418

**Published:** 2016-04-25

**Authors:** Shih-Chieh Lin, Chung-Yang Kao, Hui-Ju Lee, Chad J. Creighton, Michael M. Ittmann, Shaw-Jenq Tsai, Sophia Y. Tsai, Ming-Jer Tsai

**Affiliations:** 1 Department of Molecular and Cellular Biology, Baylor College of Medicine, Houston, Texas 77030, USA; 2 Department of Physiology, College of Medicine, National Cheng Kung University, Tainan Taiwan 701, ROC; 3 Department of Medicine, Dan L. Duncan Cancer Center Division of Biostatistics, Baylor College of Medicine, Houston, Texas77030, USA; 4 Department of Pathology and Immunology, Baylor College of Medicine, Houston, Texas 77030, USA; 5 Institute of Basic Medical Sciences, College of Medicine, National Cheng Kung University, Tainan Taiwan 701, ROC; 6 Department of Medicine and Program in Developmental Biology, Baylor College of Medicine, Houston, Texas 77030, USA

## Abstract

Although early detection and treatment of prostate cancer (PCa) improves outcomes, many patients still die of metastatic PCa. Here, we report that metastatic PCa exhibits reduced levels of the microRNAsmiR-101 and miR-27a. These micro-RNAs (miRNAs) negatively regulate cell invasion and inhibit the expression of FOXM1 and CENPF, two master regulators of metastasis in PCa. Interestingly, the repression of FOXM1 and CENPF by these miRNAs occurs through COUP-TFII, a member of the orphan nuclear receptors family. Loss of miR-101 positively correlates with the increase of COUP-TFII-FOXM1-CENPF activity in clinical PCa data sets, implicating clinical relevance of such regulation. Further studies show that COUP-TFII is a critical factor controlling metastatic gene networks to promote PCa metastasis. Most importantly, this miRNA-COUP-TFII-FOXM1-CENPF regulatory axis is also involved in the development of enzalutaminde resistance. Taken together, our findings highlight the contribution of specific miRNAs through the regulation of the COUP-TFII-FOXM1-CENPF cascade in PCa metastasis and drug resistance.

## Abstract (web summary)

The orphan nuclear receptor COUP-TFII is highly expressed in metastatic prostate cancers and its overexpression accelerates prostate tumour progression in mouse models. Here, the author show that that loss of miR-101 and miR-27a in prostate cancer cells can lead to *COUP-TFII* expression which in turn directly regulates *FOXM1* and *CENPF* favouring prostate cancer metastasis.

## Graphical abstract

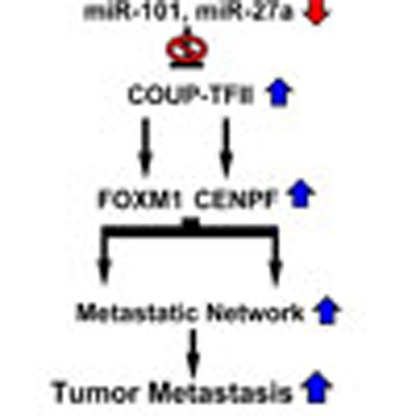

Prostate cancer (PCa) is the second commonly diagnosed cancer in men worldwide^1^. Most men with low-grade confined primary PCa are generally curable by surgery and radiotherapy^2^,^3^. However, the clinical challenge is to provide effective means for treatment of patients whose cancer advances to a deadly, late-stage, metastatic PCa. Identifying the characteristics of primary lesions that ultimately give rise to lethal metastatic phenotypes is necessary to devise innovative therapy for these patients. Although many genes and signalling pathways involved in metastasis have been reported in PCa^4^,^5^,^6^,^7^, it is still unclear how cancer cells acquire these traits.

Emerging evidence has shown that micro-RNAs (miRNAs) are involved in different stages of PCa progression including initiation, propagation and metastasis^8^. As of today, the majority of miRNA acts as tumour suppressor and its major cellular function is to inhibit cell growth, epithelial–mesenchymal transition (EMT), cell migration and invasion^9^,^10^,^11^,^12^. These findings suggest that dysregulation of miRNAs may promote PCa progression from a localized to a metastatic tumour. Therefore, identification of critical miRNAs involved in the transition from localized PCa to metastatic PCa as well as understanding their underlying molecular mechanisms would likely help us in developing better therapeutic strategies for prevention of metastasis.

COUP-TFII, an orphan nuclear receptor, has been shown to be overexpressed in a large cohort of primary PCa specimens and its expression further increased in metastatic PCa^13^. In addition, overexpression of COUP-TFII in the mouse prostate epithelium accelerates prostate tumour progression in the PTEN null prostate tumour model^13^. Importantly, molecular analysis reveals that overexpression of COUP-TFII in mice overcomes the TGF-β-induced growth barrier by interacting with SMAD4 and inhibiting SMAD4-induced TGF-β signalling in PCa^13^. These findings suggest that COUP-TFII plays an indispensable role during PCa progression. However, the potential factors causing COUP-TFII overexpression and the role of COUP-TFII in the late stage of prostate tumour metastasis have yet to be determined.

FOXM1, a forkhead domain transcriptional factor, is frequently overexpressed in different kinds of cancers, including PCa^14^,^15^. Elevated levels of FOXM1 have been shown to contribute to all major hallmarks of cancer including cellular proliferation, genomic instability, angiogenesis, metastasis and drug resistance^15^,^16^. CENPF, a structural protein of kinetochore and a known target of FOXM1, has also been shown to be upregulated and plays an important role in PCa development^17^,^18^. Recently, FOXM1 and CENPF have been identified as synergistic master regulators of PCa malignancy and as prognostic indicators of poor survival and metastasis^19^. Most intriguingly, both FOXM1 and CENPF levels are further increased in metastatic PCa^19^. These findings suggest that FOXM1 and CENPF might be critical drivers for PCa development. However, the underlying mechanism that causes dysregulation of FOXM1 and CENPF in PCa remains largely undefined.

In the present study, we identified several miRNAs, whose expressions were downregulated in PCa patients, especially in metastatic PCa. We further demonstrated that these miRNAs suppress PCa metastasis at least partially through inhibiting COUP-TFII expression, which in term directly regulates the expression of CENPF and FOXM1 as well as many genes important for PCa metastasis.

## Results

### Loss of upstream miRNA causes COUP-TFII overexpression

**Figure 1:**
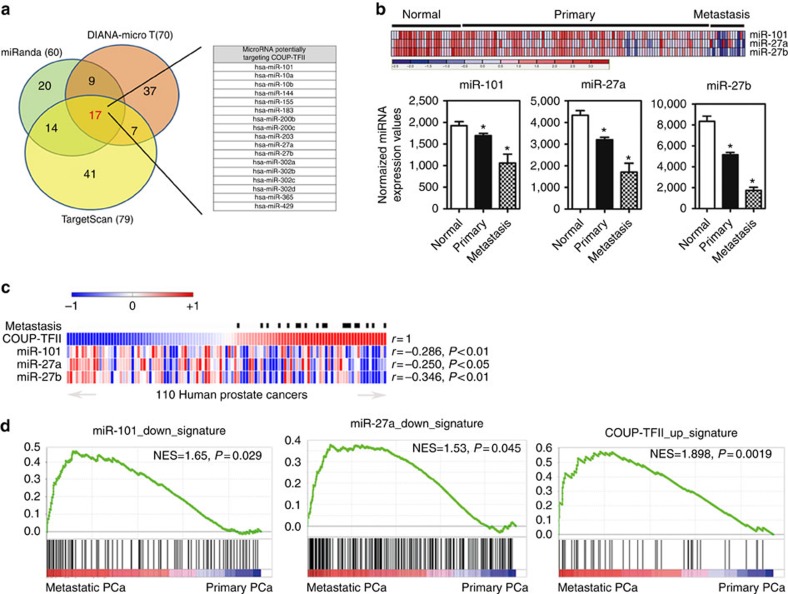
miR-101 and miR-27a loss which correlates with COUP-TFII de-repression promotes metastasis. (**a**) COUP-TFII 3′-UTR region was used for analysis of miRNA-binding sites by using three different bioinformatic tools. (**b**) Heatmap and quantified results show the levels of miR-101, miR-27a and miR-27b in the clinical PCa data set of Taylor. Normal (*n*=28); primary PCa (*n*=99); metastatic PCa (*n*=14). *P<0.05 (two-sided Student's *t*-test) compared with the normal prostate group. (**c**) Heatmap shows negative correlation between COUP-TFII and miR-101, miR-27a and miR-27b expressions in clinical PCa including primary and metastatic PCa specimens analysed from the Taylor data set. Correlation values were calculated by Pearson's correlation test. (**d**) miR-101-downregulated (GSE13674), miR-27a-downregulated (GSE65874) and COUP-TFII-upregulated (GSE33182) gene signatures were analysed by GSEA in the gene sets (GSE32269) derived from metastatic and primary patients. FDR=0.09 (miR-101); FDR=0.16 (miR-27a); and FDR=0.02 (COUP-TFII).

Recently, a number of miRNAs have been implicated to play crucial roles during prostate tumour progression; however, there is no systematic analysis of their function in prostate tumour metastasis at present. Since the underlying mechanism for the upregulation of COUP-TFII expression in PCa is not known, we ask whether dysregulation of miRNA is the potential reason causing COUP-TFII overexpression and promoting metastasis in PCa. To test this hypothesis, we used three different bioinformatic prediction tools to analyse if miRNA-binding sites are present in the COUP-TFII 3′-untranslated region (UTR) region and identified 17 miRNAs-binding sites (Fig. 1a). To assess the potential clinical relevance of these miRNAs that targeted COUP-TFII to impact metastatic PCa, we used an *in silico* approach to systematically analyse the changes in levels of miRNAs in metastatic PCa employing two different public miRNA data sets (GSE21036 and GSE26964) and used a criteria of false discovery rate (FDR) <0.05 and more than twofold change. As shown in the Supplementary Table 1, 31 downregulated miRNAs and two upregulated miRNAs were identified in metastatic PCa. Among these miRNAs, miR-101, miR-27a and miR-27b have putative binding sites at the 3′-UTR region of COUP-TFII mRNA as indicated in Fig. 1a. We then re-analysed the expression profiles of miR-101, miR-27a and miR-27b in the Taylor data set^20^, which contains normal and PCa tissues. Heatmap and quantified results indicated that the levels of miR-101, miR-27a and miR-27b were reduced in the primary PCa samples and the expressions were further reduced in the metastatic PCa samples (Fig. 1b). Most importantly, COUP-TFII levels were negatively correlated with miR-101, miR-27a and miR-27b levels in clinical PCa specimens (Fig. 1c). This result suggests that the decreased expression of miR-101, miR-27a and miR-27b might contribute to the observed overexpression of COUP-TFII in PCa patients. If regulation of COUP-TFII by miR-101, mR-27a and miR-27b is indeed important for PCa metastasis, we expect that these miRNA levels will have a strong negative correlation with COUP-TFII levels in metastatic PCa patients. Indeed, there were strong negative correlations between COUP-TFII, miR-101 and miR-27a, but not miR-27b (Fig. 1c and Supplementary Fig. 1) in metastatic PCa specimens. In addition, Gene Set Enrichment Analysis (GSEA) of the molecular signatures' database indicated that downstream target genes of miR-101, miR-27a as well as COUP-TFII mRNA and its signatures were significantly depleted (miR-101 and miR-27a) or enriched (COUP-TFII) in the metastatic PCa patient (Fig. 1d). Therefore, we focus our study on the roles of the miR-101, and miR-27a in regulating COUP-TFII expression in PCa metastasis.

### COUP-TFII is negatively regulated by miR-101 and miR-27a

**Figure 2:**
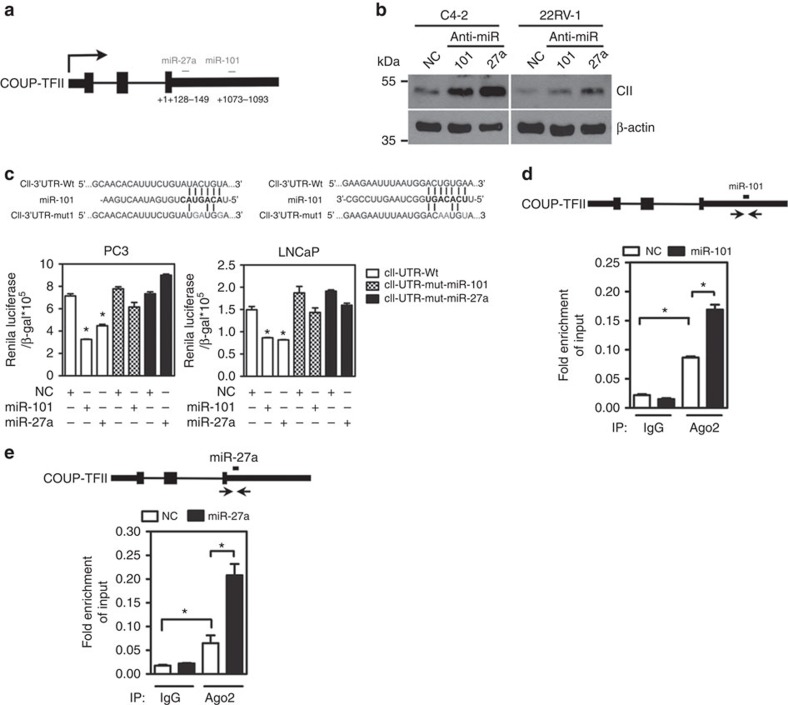
The expression of COUP-TFII is repressed by miR-101 and miR-27a. (**a**) Cartoon indicates the recognition sites for miR-101 and miR-27a. (**b**) Representative western blot demonstrated the levels of COUP-TFII in 22RV-1 cells treated with inhibitors of miR-101 and miR-27a for 72 h. (**c**) WT- and mutated- miR-101 and miR-27a recognition sites are shown in the upper panel. COUP-TFII 3′-UTR promoter activity was performed in LNCaP and PC3 cells overexpressing miR-101 and miR-27a mimics for 48 h (*n*=3). *P<0.05 (two-sided Student's *t*-test) compared with the NC (scramble control) group. (**d**,**e**) A cartoon shows location of primers in the COUP-TFII 3′-UTR region (upper panel). Levels of COUP-TFII 3′-UTR regions containing miR-101 and miR-27a-binding sites that were immunoprecipitated with Ago2 or IgG antibody from PC3 cells after being treated with miR-101 (**d**) or miR-27a (*n*=3). (**e**) mimic for 72 h. *P<0.05 (two-sided Student's *t*-test).

To assess whether miR-101 and miR-27a regulate COUP-TFII expression through their putative binding sites located in the 3′-UTR region of COUP-TFII mRNA (Fig. 2a), we screened the expression levels of COUP-TFII in normal epithelial and PCa cell lines first (Supplementary Fig. 2A). Since the expression of COUP-TFII is low in C4-2 and 22RV-1 cells, we asked whether blockade of endogenous miR-101 and miR-27a by inhibitors (antisense RNA) in these cells could increase COUP-TFII expression. As shown in Fig. 2b, indeed inhibition of endogenous miR-101 and miR-27a markedly increased COUP-TFII expression. In contrast, overexpression of miR-101 and miR-27a significantly reduced COUP-TFII levels (Supplementary Fig. 2B) in LNCaP and PC3 cells, which have higher COUP-TFII expressions. Most importantly, miR-101 and miR-27a have at least additive effects on inhibition of COUP-TFII expression (Supplementary Fig. 2C,D).

To further evaluate the function of putative binding sites in the COUP-TFII 3′-UTR region, we inserted the wild-type (WT) or mutated COUP-TFII 3′-UTR region into a reporter construct and performed reporter assays using LNCaP and PC3 cells. Figure 2c shows that overexpression of either miRNAs significantly reduced reporter activity in the WT COUP-TFII 3′-UTR construct. However, the inhibitory effects exerted by miR-101 and miR-27a were abrogated when their binding sites were mutated. These results suggest that miR-101 and miR-27a work through these sites to control COUP-TFII expression. To further investigate whether these miRNAs interact with COUP-TFII mRNA inside the cell, PC3 cells were first treated with miR-101 mimic before Ago2-RNA-immunoprecipitation (RIP) assay was carried out. Ago2 is a component of the RNA-induced silencing complex which forms at the miRNA inhibiting sites. Thus, we designed a primer to amplify the region containing miR-101-binding sites within the COUP-TFII 3′-UTR region (Fig. 2d). Real-time quantitative PCR (RT-qPCR) analysis shows that Ago2 is present at the COUP-TFII 3′-UTR and overexpression of miR-101 further increased the interaction between Ago2-miR-101 complexes and COUP-TFII mRNA (Fig. 2d). Similar results were also observed for miR-27a (Fig. 2e). These results suggest that miR-101 and miR-27a interact with COUP-TFII mRNA at the 3′-UTR binding site in PCa cells.

### Loss of miRNA promotes metastasis by inducing COUP-TFII

**Figure 3:**
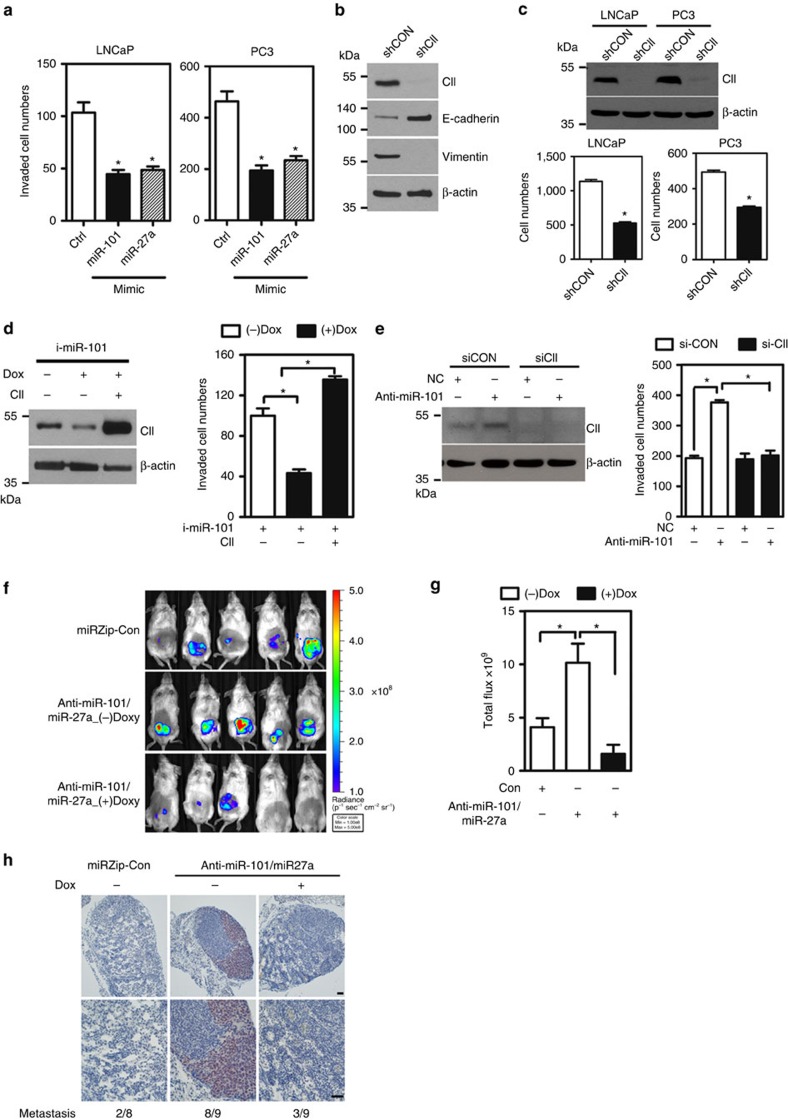
Loss of miR-101 promotes cancer metastasis through de-repression of COUP-TFII expression. (**a**) LNCaP and PC3 cells were individually treated with 50 nM of miR-101 and miR-27a mimic for 48 h and then invasion assays were performed for an additional 16 h (*n*=3). *P<0.05 (two-sided Student's *t*-test) compared with the control group. (**b**) Representative western blot showed the levels of COUP-TFII, E-cadherin, vimentin and β-actin in PC3 cells stably knocked down by COUP-TFII. (**c**) LNCaP and PC3 cells stably knocked-down of COUP-TFII were used to perform cell invasion assay. Invaded cells were stained and counted (lower panel) (*n*=3). Representative western blot showed the knockdown efficiency of COUP-TFII (upper panel). *: *P*<0.05 (two-sided Student's *t*-test) compared with control group. (**d**) PC3 cells carrying an inducible miR-101 in the absence and presence of doxycycline, and re-expression of COUP-TFII were used to perform invasion assays (*n*=3). Representative western blot showed the levels of COUP-TFII and β-actin (left). Invaded cells were counted and results are shown in the right panel. *: *P*<0.05 (two-sided Student's *t*-test) (**e**) 22RV-1 cells were treated with miR-101 inhibitor (antisense RNA) in conjunction with knockdown of COUP-TFII and used for invasion assays (*n*=3). Representative western blot show the levels of COUP-TFII and β-actin (left). Invaded cells were counted and result is shown in the right panel. *: *P*<0.05 (two-sided Student's *t*-test). (**f**) LNCaP cells containing a construct with inducible expression of COUP-TFII shRNA and constructs expressing with anti-miR-101 and anti-mir-27a were orthotopically injected into NOD-SCID mouse prostate. In addition these cells also contain a luciferase reporter to detect cancer cells. After the tumour size was bigger than 50 mm^3^, drinking water with or without doxycycline (1 mg ml^−1^) was given to the mice for 6 weeks to induce the COUP-TFII shRNA to repress COUP-TFII expression. Representative bioluminescence results show the status of tumour growth in different groups. (**g**) Quantification result of luminance from IVIS (control: *n*=5; anti-miR-101/27a(−): *n*=6; anti-miR-101/27a(+): *n*=7. *: *P*<0.05 (two-sided Student's *t*-test). (**h**) Immunohistochemical stain showed the metastatic tumour cells located in the mouse lymph node are positive for AR. Scale bar: 100 μM (low power); and 200 μM (high power). SCID, severe combined immunodeficient.

Analysis of miR-101, miR-27a and COUP-TFII signatures revealed their potential roles in metastatic PCa. Since COUP-TFII is shown to be the downstream target of miR-101 and miR-27a, we hypothesize that miR-101 and miR-27a may regulate metastasis through modulation of COUP-TFII expression. To investigate whether miR-101 and miR-27a are involved in the metastatic process, we first performed gain- and loss-of function experiments using *in vitro* migration and invasion assays. Results demonstrate that overexpression of miR-101 and miR-27a in LNCaP and PC3 cells significantly inhibit cell migration and invasion (Supplementary Fig. 3A and Fig. 3a), while knockdown of those miRNAs promote cell migration and invasion (Supplementary Fig. 3B,C). These results are consistent with our previous findings that overexpression of COUP-TFII promotes PCa metastasis by inhibiting the TGF-β-induced barrier^13^. Here, we further found that COUP-TFII is important for EMT since knockdown of COUP-TFII led to induction of E-cadherin and a reduction of vimentin expression (Fig. 3b). Furthermore, loss of EMT is reflected by the reduction of cell invasion (Fig. 3c), suggesting a critical role for COUP-TFII in EMT and cell invasiveness. To ascertain that upstream miRNA indeed negatively regulates COUP-TFII expression to inhibit cell invasion, we used the doxycycline (Dox) inducible system to stably induce the expression of miR-101 in PC3 cells. Western blot indicated that induction of miR-101 levels decreased COUP-TFII expression and impeded cell invasion (Fig. 3d). Upon ectopic re-expression of COUP-TFII, cell invasion ability was restored (Fig. 3d). Conversely, treatment with miR-101 inhibitor enhanced COUP-TFII expression and thus promoted cell migration (Supplementary Fig. 4A) and cell invasiveness (Fig. 3e), while knockdown of COUP-TFII by siRNA-eliminated cell invasiveness induced by the downregulation of miR-101 (Fig. 3e). Similar results were shown when the functions of both miR-101 and miR-27a were blocked in LNCaP cells (Supplementary Fig. 4B). These *in vitro* results suggest that effects of cell invasiveness by miRNAs are in part through regulating COUP-TFII expression. To directly test whether miRNA-regulated COUP-TFII levels are functionally important for PCa metastasis *in vivo*, inducible knockdown of COUP-TFII in LNCaP cells carrying anti-miR-101 and anti-miR-27a constructs were orthotopically injected into mouse prostate. Results demonstrated that inhibition of both miR-101 and miR-27a not only markedly increased tumour growth but also promoted lymphatic metastasis as indicated by androgen receptor (AR) or GFP staining, confirming that metastatic loci in the mouse lymph node came from LNCaP cells (Fig. 3f–h and Supplementary Fig. 4C). Most importantly, knockdown of COUP-TFII abrogated anti-miRNA affects tumour growth and lymphatic metastasis (Fig. 3f–h). Similar results employing PC3 cells expressing anti-miR-101 and anti-miR-27a markedly accelerated tumour growth and promoted lymphatic metastasis through the usage of the orthotopic injection mouse model with GFP as a marker for injected cells (Supplementary Fig. 5). Taken together, these results suggest that loss of upstream miRNA results in metastasis of PCa through de-repression of COUP-TFII expression.

### COUP-TFII inducing FOXM1 and CENPF to promote metastasis

**Figure 4:**
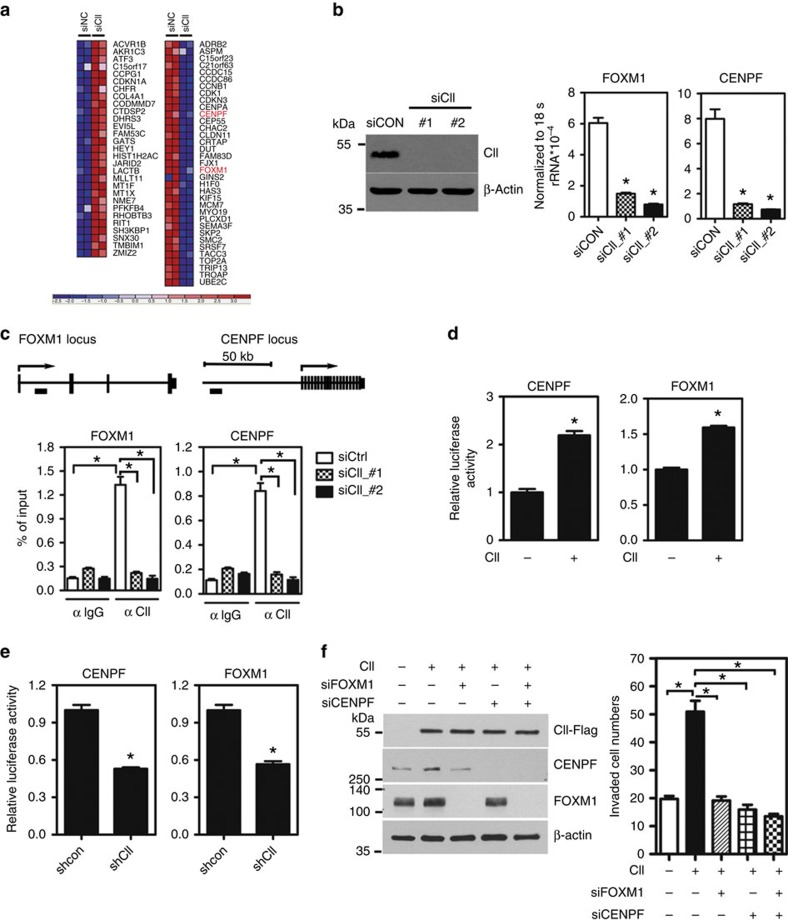
FOXM1 and CENPF are downstream targets of COUP-TFII in PCa. (**a**) Gene expression profile of COUP-TFII knockdown PC3 cells (GSE33182) was downloaded from GEO database and analysed using GenePattern. Genes with *P*<0.05, FOLD CHANGE >1.5-fold are represented as a heatmap. The gene symbol of *FOXM1* and *CENPF* genes after knockdown of COUP-TFII are annotated in red colour. The others were FOXM1 and CENPF co-regulated genes annotated in black colour. siCtrl: knockdown scramble control; siCII: knockdown of COUP-TFII. (**b**) A representative western blot shows the level of COUP-TFII in PC3 cells transfected with two different siRNAs against COUP-TFII for 72 h (upper panel). Levels of FOXM1 and CENPF are quantified by RT-qPCR (lower panel) (*n*=3). *P<0.05 (two-sided Student's *t*-test) compared with the control group (siCON). (**c**) A cartoon shows the FOXM1 and CENPF loci. COUP-TFII-binding sites are annotated by a black rectangle (upper panel). ChIP results are shown in the PC3 cells treated with control and two different siRNAs against COUP-TFII for 72 h (lower panel) (*n*=3). *P<0.05 (two-sided Student's *t*-test) compared with control. (**d**) FOXM1 and CENPF promoter activities were measured in PC3 cells carrying an inducible *COUP-TFII* gene in the absence or the presence of doxycycline for 48 h (*n*=3). *P<0.05 (two-sided Student's *t*-test). (**e**) FOXM1 and CENPF promoter activities were measured in control or stably knocked-down COUP-TFII in PC3 cells for 48 h (*n*=3). *P<0.05 (two-sided Student's *t*-test). (**f**) PC3 cells carrying with inducible COUP-TFII-Flag gene were treated with or without doxycycline for 48 h and then transfected with individual siRNA against FOXM1 and CENPF or double knockdown of FOXM1 and CENPF for an additional 48 h. western blot (left panel) shows the level of COUP-TFII, CENPF and FOXM1 expression. Invasion assay (right panel) was performed by using those cells (*n*=3). *P<0.05 (two-sided Student's *t*-test) compared with control.

Recently, FOXM1 and CENPF have been identified as critical drivers in PCa progression in both human and in mouse, and their co-expression is a prognostic indicator of poor survival and metastasis^19^. Most importantly, their expression levels are further elevated in metastatic PCa from different public data sets^19^. However, the upstream factor that controls the expression of FOXM1 and CENPF has yet to be identified. Here, we found that knockdown of COUP-TFII significantly reduced the expressions of FOXM1, CENPF and their downstream target genes in our previous microarray data (Fig. 4a), suggesting that FOXM1 and CENPF are likely downstream of COUP-TFII. To elucidate how COUP-TFII regulates FOXM1 and CENPF expressions to promote metastasis, we knocked-down COUP-TFII by two different siRNAs in PC3 cells and detected a significant reduction of the expression levels of FOXM1, CENPF and consequently their downstream targets (Fig. 4b and Supplementary Fig. 6A). Similar results were also observed in LNCaP cells (Supplementary Fig. 6B). In contrast, upregulation of COUP-TFII expression increased their expressions in PC3 cells (Supplementary Fig. 7A,B). Finally, we asked whether COUP-TFII regulates these target genes through FOXM1 and CENPF. For this purpose, we engineered PC3 cells with Dox inducible expression of COUP-TFII. Using these cells, we found that indeed COUP-TFII regulates these genes' expression through FOXM1 and CENPF (Supplementary Fig. 6C).

To further evaluate whether COUP-TFII directly regulates FOXM1 and CENPF levels, we checked our previous COUP-TFII chromatin-immunoprecipitation (ChIP)-seq data (Supplementary Fig. 7C,D) and found that COUP-TFII-binding sites were located in the gene locus of FOXM1 and CENPF. Using ChIP-qPCR analysis, we further demonstrated robust recruitment of COUP-TFII to its binding sites on the FOXM1 and CENPF promoter in comparison with the IgG control (Fig. 4c). In contrast, knockdown of COUP-TFII by two different siRNAs largely abolished its recruitment. These results indicate that COUP-TFII is recruited to the promoter to directly regulate the expression of these two genes. To further substantiate this conclusion, luciferase reporter assays were carried out to show that expression of COUP-TFII can indeed stimulate FOXM1- and CENPF-promoter driven reporter activities (Fig. 4d), while knockdown of COUP-TFII reduced their promoter activities (Fig. 4e). These results strongly support the conclusion that COUP-TFII is recruited to the promoter of FOXM1 and CENPF to regulate their expression at the transcriptional level. Since COUP-TFII positively regulates FOXM1 and CENPF levels, we further examined whether COUP-TFII promotes cell invasion through FOXM1 and CENPF. For this purpose, we stably induced COUP-TFII expression in PC3 cells. Induction of COUP-TFII increased FOXM1 and CENPF expression and enhanced cell invasion (Fig. 4f). However, knockdown of FOXM1 or CENPF by itself or knockdown of both together abrogated COUP-TFII-induced cell invasion (Fig. 4f). Collectively, these results indicate that COUP-TFII-induced cell invasion is largely mediated by the induction of FOXM1 and CENPF expression.

### miRNA represses FOXM1/CENPF level via COUP-TFII

**Figure 5:**
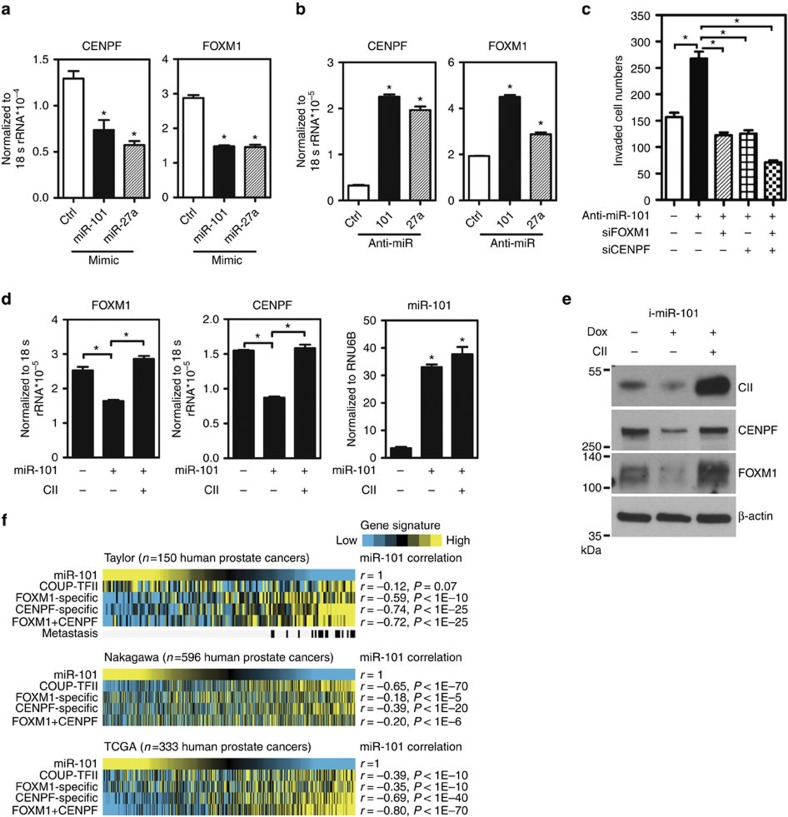
miR-101-inhibited COUP-TFII leads to FOXM1 and CENPF downregulation in PCa. (**a**,**b**) Levels of CENPF and FOXM1 in LNCaP cells having overexpression of miR-101and miR-27a for 72 h (*n*=3) (**a**) and in 22RV-1 stably transfected with control, anti-miR-101 or miR-27a vectors (*n*=3) (**b**). *P<0.05 (two-sided Student's *t*-test) compared with the control group. (**c**) 22RV-1 cells treated with 50 nM miR-101 inhibitor and 25 nM siRNA against FOXM1 or CENPF were plated into invasion chamber for 16 h (*n*=3). Invaded cell numbers were stained and counted. *P<0.05 (two-sided Student's *t*-test) compared with the control group. RT-qPCR (**d**) and representative western blot (**e**) shows the levels of FOXM1, CENPF and miR-101 from PC3 cells carrying an inducible *miR-101* gene in the absence and presence of doxycycline (*n*=3). Also shown in this figure is the rescue of FOM1 and CENPF expression when COUP-TFII is ectopically expressed for 96 h. *P<0.05 (two-sided Student's *t*-test) (**f**) miR-101, COUP-TFII, FOXM1, CENPF and FOXM1-CENPF co-regulated gene signatures were analysed in primary PCa specimens for three different expression data sets of human tumours. Yellow, high-signature scoring in prostate tumour specimens, is indicative of high manifestation of associated transcriptional patterns; blue, equals low-signature scoring. Correlation between miR-101 signature scores and scores for other signatures were tested by Pearson's correlation.

Since miR-101 and miR-27a directly inhibited COUP-TFII expression, and FOXM1 and CENPF were downstream targets of COUP-TFII, we hypothesized that repression of FOXM1 and CENPF expression by miR-101 and miR-27a might be mediated via COUP-TFII. To test this hypothesis, we first analysed the correlation between miR-101, miR-27a, FOXM1 and CENPF expressions in clinical PCa specimens. Results show that the levels of FOXM1 and CENPF individually had negative correlation with miR-101 and miR-27a expressions (Supplementary Fig. 8A). Moreover, overexpression of miR-101, and miR-27a reduced FOXM1 and CENPF expressions in LNCaP cells (Fig. 5a). In contrast, inhibition of miR-101 and miR-27a increased the levels of FOXM1 and CENPF in 22RV-1 cells (Fig. 5b). In addition, downregulation of FOXM1 and CENPF had no effect on miRNA expression (Supplementary Fig. 8B). Next, we asked whether FOXM1 and CENPF are responsible for miRNA-mediated inhibition of cell invasion. For this purpose, we used 22RV-1 cells stably overexpressed an anti-miR-101 construct and manipulated the expression of FOXM1 and CENPF through their siRNAs and then performed cell invasion assay. Cell invasion increased when miR-101 was inhibited (Fig. 5c). Such induction of cell invasion was abolished by either individual or double knockdown of FOXM1 and CENPF expression (Fig. 5c). Next, to address whether COUP-TFII mediates miRNAs inhibition of FOXM1 and CENPF expression, we used inducible-miR-101 stable clone in PC3 cells to assess whether restoring the expression of COUP-TFII in miR-101 overexpressed cells could rescue the expressions of FOXM1 and CENPF. Results obtained from RT-qPCR (Fig. 5d) and western blot (Fig. 5e) analyses clearly show that inhibition of expression of FOXM1 and CENPF by miR-101 was nullified with re-expression of COUP-TFII. Similar results were shown using an inducible-miR-27a stable clone (Supplementary Fig. 9). Finally, to demonstrate the clinical relevance of the miRNA-COUP-TFII-CENPF-FOXM1 regulation cascade, we analysed their signatures in three large cohorts of clinical PCa data sets. As shown in Fig. 5f, using three different clinical PCa data sets, we found that the miR-101 signature had significantly negative correlation with COUP-TFII, CENPF and FOXM1 signatures in all the data sets analysed. Similar results were shown using miR-27a signature (Supplementary Table 2). Furthermore, this negative correlation was more obvious in the PCa specimens with metastasis (Fig. 5f). Finally, we also demonstrated that regulation cascade of miR-101-COUP-TFII-CENPF-FOXM1 is a general phenomenon occurring in many cancer types, not only in PCa (Supplementary Fig. 10). Most importantly, COUP-TFII, miR-101 and miR-27a were good indicators for predicting progression of malignant PCa (Supplementary Fig. 11 and Supplementary Table 3). The expression level of COUP-TFII is positively correlated with the Gleason score of PCa and with metastasis of prostate, colon and breast cancer. Taken together, these results reveal that loss of miR-101 or miR-27a expression during PCa progression results in the upregulation of COUP-TFII expression, which in turn upregulates the expression of two of the most important oncogenes in PCa, FOXM1 and CENPF.

### COUP-TFII is a master regulator of the metastatic network

**Figure 6:**
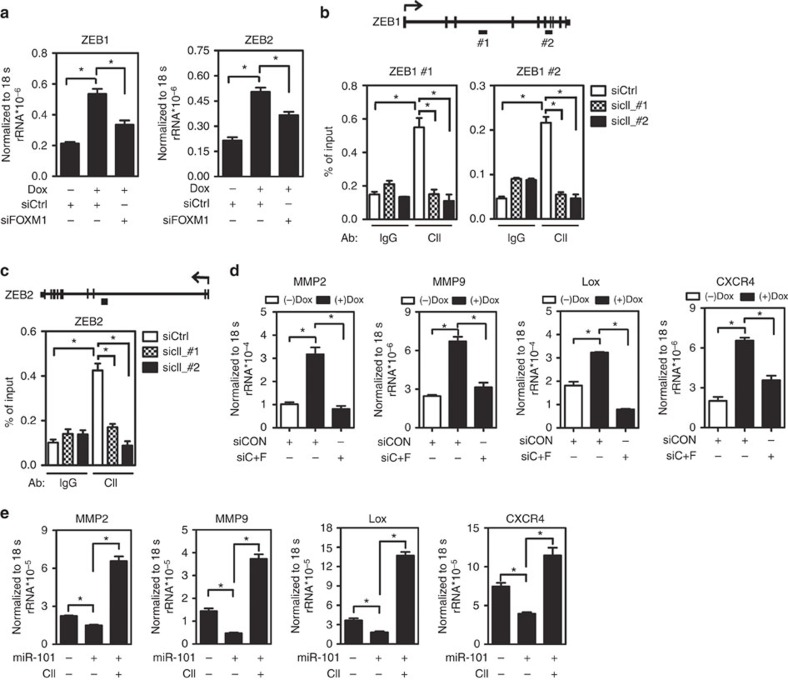
COUP-TFII is a master regulator central to the metastatic gene regulatory network. (**a**) Levels of ZEB1 and ZEB2 expression in PC3 cells carrying an inducible *COUP-TFII* gene expression construct were determined after 72 h treatment in the absence or the presence of doxycycline and FOXM1 siRNA (*n*=3). *P<0.05 (two-sided Student's *t*-test). (**b**,**c**) A cartoon shows the ZEB1 and ZEB2 loci. COUP-TFII-binding sites as annotated by a black rectangle (upper panel). ChIP quantification results are shown in the PC3 cells treated with control or two different siRNAs against COUP-TFII for 72 h (lower panel) (*n*=3). *P<0.05 (two-sided Student's *t*-test) compared with control group. (**d**) Levels of MMP2, MMP9, LOX and CXCR4 in PC3 cells carrying an inducible *COUP-TFII* gene in the absence or the presence of doxycycline, and subsequent knock down of both CENPF and FOXM1 expression (siC+F) for 72 h (*n*=3). *P<0.05 (two-sided Student's *t*-test). (**e**) Levels of MMP2, MMP9, LOX and CXCR4 in PC3 cells carrying an inducible *miR-101* gene in the absence or the presence of doxycycline, and subsequent enforced expression of COUP-TFII for 96 h (*n*=3). *P<0.05 (two-sided Student's *t*-test).

To further investigate the potential mechanisms of how FOXM1 and CENPF mediated COUP-TFII function to promote tumour metastasis, we specifically constructed a metastasis gene regulatory network in PCa using neoplasm metastasis-related genes classified by MetaCore and crossing them with the gene list changed in the metastatic PCa (Supplementary Fig. 12). On the basis of the well-known function of FOXM1 in metastasis, we selected some FOXM1 downstream targets, including two important EMT transcriptional factors, ZEB1 and ZEB2, and four effectors (matrix metalloproteinases 2 [MMP2], matrix metalloproteinases 9 [MMP9], chemokine lysyl oxidase [LOX] and (C-X-C Motif) receptor 4 [CXCR4] as readouts to test whether COUP-TFII could control the expression of the FOXM1 downstream target genes. Both mRNAs for ZEB1 and ZEB2 were decreased upon knockdown of COUP-TFII (Supplementary Fig. 13A). In parallel, overexpression of COUP-TFII increased their expression (Supplementary Fig. 13B). Since COUP-TFII-induced ZEB1 and ZEB2 expression did not completely abolish with knockdown of FOXM1 expression (Fig. 6a), we suspected that COUP-TFII may regulate ZEB1 and ZEB2 by direct transcriptional regulation. Indeed, COUP-TFII was recruited to promoter/enhancer regions of ZEB1 and ZEB2 to induce their expression (Fig. 6b,c).

MMP2, MMP9, LOX and CXCR4 play critical roles in metastasis via degradation of the extracellular matrix, formation of the pre-metastatic niche and homing to the bone marrow, respectively^21^,^22^,^23^,^24^,^25^,^26^,^27^. To further investigate whether these genes are regulated by COUP-TFII, we showed that knockdown of COUP-TFII significantly reduced MMP2, MMP9, LOX and CXCR4 expressions (Supplementary Fig. 13C). Conversely, overexpression of COUP-TFII markedly increased their expression (Supplementary Fig. 13D) and knockdown of FOXM1 and CENPF abrogated COUP-TFII effect on these genes' expression (Fig. 6d). Furthermore, the repressions of these gene expressions by miR-101 were lost upon re-expression of COUP-TFII (Fig. 6e). In contrast, inhibition of both miRNA-101 and miR-27a expression were shown to increase the expression of MMP2, MMP9, LOX and CXCR4 and downregulation of COUP-TFII expression abolished this increase (Supplementary Fig. 14). Taken together, our results support the notion that loss of upstream miRNA enhances the expression of COUP-TFII, which serves as a master regulator to orchestrate a metastatic network in PCa.

### miRNA-COUPTFII-CENPF-FOXM1 promotes drug resistance

**Figure 7:**
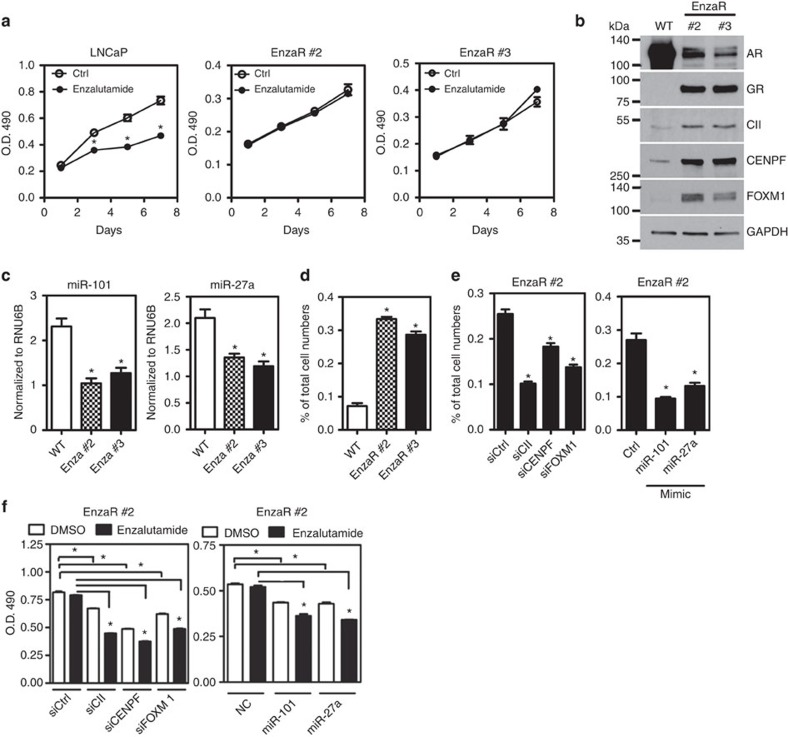
miRNA-COUP-TFII-CENPF-FOXM1 regulation axis contributes to the enzalutamide resistance. (**a**) Parental cell (LNCaP) and enzalutamide-resistant (EnzaR) clones were treated with 10 μM enzalutamide for indicated time point and cell growth was measured by MTS assay (*n*=3). *P<0.001 (two-way ANOVA test). (**b**) A representative western blot shows the expression of COUP-TFII, CENPF, FOXM1, AR and GR (glucocorticoid receptor), a positive control gene for enzalutamide-resistant clones^44^. (**c**) RT-real-time PCR data demonstrated the expressions of miR-101 and miR-27a in LNCaP (WT) and EnzaR clones (*n*=3). *P<0.05 (one-way ANOVA followed by Dunnett test). (**d**) LNCaP (WT) and EnzaR cells were used to perform migration chamber assay for 16 h (*n*=3). Then, invaded cells were counted and normalized to total cell number. *P<0.0001 (one-way ANOVA followed by Dunnett test) (**e**,**f**) Knockdown of COUP-TFII, CENPF or FOXM1, or overexpression of miR-101 or miR-27a in enzalutamide clone (#2) was used to perform migration chamber assay for 16 h (*n*=3). *P<0.0001 (one-way ANOVA followed by Dunnett test) (**e**) or measure cell growth by MTS assay after treating with or without 10 μM enzalutamide for 3 days (**f**). *P<0.0001 (one-way ANOVA followed by Dunnett test). ANOVA, analysis of variance.

Recently, resistance for enzalutamide (a second-generation anti-androgen drug, also called MDV-3100) is a critical issue in clinical therapy^28^. To answer whether androgen-deprivation therapy would affect the regulation cascade of miRNA-COUPTFII-CENPF-FOXM1, we selected several enzalutamide-resistant clones by treating LNCaP cells with 10 μM enzalutamide for at least 3 months. Results show that enzalutamide markedly inhibited the growth of LNCaP cells (parental cells), but has no effect on enzalutamide-resistant (EnzaR) clones (Fig. 7a). Using these cells, we found that COUP-TFII, CENPF and FOXM1 were markedly increased, while miR-101 and miR-27a expression significantly decreased in EnzaR clones compared with parental cells (Fig. 7b,c). Next, we investigated the migration ability in EnzaR clones and their parental cells. Interestingly, results showed that EnzaR clones have higher migration ability compared with their parental cells (Fig. 7d). To further investigate the roles of the miRNA-COUPTFII-CENPF-FOXM1 regulation cascade, we knocked down COUP-TFII, CENPF or FOXM1 expression or overexpressed miR-101 or miR-27a in EnzaR clones. Results revealed that overexpression of miR-101 or miR-27a, and knockdown of COUP-TFII, CENPF or FOXM1 not only reduced migration ability (Fig. 7e) but also increased efficacy of enzalutamide treatment (Fig. 7f) in enzalutamide-resistant clones. Taken together, these results indicate that regulation of the miRNA-COUPTFII-CENPF-FOXM1 cascade may promote the development of enzalutamide resistance in PCa. Thus, targeting this regulation cascade may provide alternative therapeutic means to alleviate enzalutamide resistance.

## Discussion

Clinically, metastatic PCa remains an incurable disease. Although numerous mediators of metastasis have been identified in PCa, these factors are generally difficult to target. Recent advancement in microRNA (miRNA) -based therapy has rendered it as a more feasible way to target cancer and various delivery strategies have since been developed^29^,^30^,^31^. Therefore, it is important to identify the critical miRNAs that are associated with the metastatic process of PCa, so that efficacious therapeutic agents could be appropriately tested. Because of the above reasons, the goal of our current study is to identify the critical miRNAs that targeted COUP-TFII and impacted on the metastatic process of PCa.

Earlier studies commonly used PCa cell lines or small cohorts of PCa specimens without metastatic PCa to identify the candidate miRNAs involved in metastasis^32^,^33^,^34^,^35^,^36^. Therefore, results from previous miRNA profiling are frequently controversial and difficult to identify the miRNAs that impact PCa metastasis. Whether those miRNAs truly have clinical relevance needs to be further validated before they can become useful therapeutic targets. Here, we analysed miRNA targeting sites located in the COUP-TFII 3′-UTR region using three different bioinformatic tools and combined analytical results of miRNA expression profile in a large cohort of PCa specimens containing normal, localized and metastatic tissues from the Taylor data set^20^ to identify clinical relevance of miRNA targeted to COUP-TFII in PCa. Our results demonstrated that loss of miR-101 and miR-27a expressions in the PCa specimens, especially in metastatic PCa, de-repressed the expression of their downstream targets and significantly augmented the metastatic phenotype. These findings suggest that loss functions of miR-101 and miR-27a may play critical roles in PCa metastasis. To further verify their roles in PCa metastasis, we showed that these miRNA not only negatively regulated PCa cell invasion ability but also COUP-TFII expression through binding to the miRNA recognition sites located in the COUP-TFII 3′-UTR region.

Recently, FOXM1 and CENPF have been reported as master regulators in human and mouse PCa using cross-species computational analysis, and their higher levels can be used as a prognostic indicator for poor outcome and metastasis^19^. However, the factors that cause dysregulation of FOXM1 and CENPF in PCa remain elusive. Here, we demonstrated that FOXM1 and CENPF were not directly regulated by miR-101 and miR-27a since there was no miR-101 and miR-27a-binding sites identified in their 3′-UTR regions. Instead, FOXM1 and CENPF are direct targets of COUP-TFII whose expression is suppressed by miR-101 and miR-27a. Thus, the expression of FOXM1 and CENPF is indirectly regulated by miR-101 and miR-27a through COUP-TFII. Most importantly, we showed that the miRNA-COUP-TFII-CENPF-FOXM1 regulatory cascade was clearly evident in clinical PCa specimens as revealed by the close correlation between their gene signatures in PCa patients. In addition, to address whether this regulatory cascade is prostate specific or a general phenomenon, we performed signature correlation analysis in other cancer types. Results demonstrate that miR-101-COUP-TFII-CENPF-FOXM1 regulation cascade is not specific to PCa, but it also occurs in many other cancer types. Taken together, to our knowledge, this is the first report that shows how both FOXM1 and CENPF are dysregulated and overexpressed in PCa.

Metastasis is a multiple-step process that includes intravasation, circulation, extravasation and colonization^37^. Many gene products and signalling pathways involved in different steps have been reported^37^. However, most studies focused on a single gene function in a particular step of metastasis, making it difficult to formulate a comprehensive gene network that impacts on metastasis. A particular interesting finding in this study is that we compared genes changed in the clinical metastatic PCa with metastasis-related genes classified by MetaCore to construct the metastatic gene network specifically in PCa. We found that COUP-TFII promoted EMT transition through both direct and indirect regulation of the expression of ZEB1 and ZEB2, which are downstream targets of FOXM1 (refs 38, 39). In addition, COUP-TFII also positively regulates genes important for metastasis, including MMP2, MMP9, LOX, CXCR4 and CXCL12. MMP2 and MMP9 are matrix metalloproteinases responsible for degradation of the extracellular matrix, and their expressions correlate with PCa metastasis^21^,^22^. LOX, a copper dependent amine oxidase, can promote collagen crosslinking at pre-metastatic organs to form a receptive niche for arriving tumour cells^26^,^27^. The CXCR4/CXCL12 axis is known to play a major role in haematopoietic stem cell (HSC) homing to the bone marrow, normally. Interestingly, CXCR4 levels are significantly increased in PCa specimens, especially in metastatic PCa^24^. Recent studies further demonstrated that disseminated PCa cells can target to the HSC niche and compete with HSC cells for the niche via the CXCR4/CXCL12 axis to facilitate metastasis^5^. Taken together, COUP-TFII, sitting on the top of the regulatory network, could be considered a pivotal factor important for promoting PCa metastasis through a diverse signalling cascade.

Androgen-deprivation therapy is the mainstream treatment strategy for PCa patients. Although it is effective to suppress tumour progression in the majority of PCa patients, most of them eventually develop hormone resistant PCa. This aggressive and incurable disease is considered as castration-resistant PCa (CRPC). Within the CRPC group, the majority of patients (∼90%) will finally develop bone metastasis which is called metastatic CRPC (mCRPC)^40^. Since CRPC often remains dependent on AR signalling, there are several second-generation anti-androgen drugs that are approved by the US Food and Drug Administration (FDA), such as enzalutamide (also called MDV3100)^41^. Enzalutamide can bind to the ligand binding domain of the AR and prevent translocation of AR into the nucleus. It can improve overall survival of men with mCRPC^41^,^42^. However, many CRPC patients who are initially responsive to the enzalutamide treatment acquire resistance to this second-generation drug^28^. Recently, AR mutation^43^ and induction of glucocoticoid receptor (GR), which can bypass androgen signalling^44^, have been proposed to be the underlying mechanisms contributing to enzalutamide resistance. Here, we provide an alternative mechanism involved in enzalutamide resistance through dysregulation of the miRNAs-COUP-TFII-FOXM1-CENPF axis. Furthermore, re-expression of those miRNAs or repression of COUP-TFII expression can reduce migration ability and increase enzalutamide efficacy to the resistant cells. Thus, upregulation of these miRNAs and downregulation of the COUP-TFII-FOXM1-CENPF axis could be combined with enzalutamide treatment to increase its therapeutic efficacy.

In conclusion, we showed that loss of function of miR101 and miR27a as revealed by the clinical PCa data set, not only leads to overexpression of COUP-TFII, FOXM1 and CENPF but also enhances PCa metastasis and drug resistance. Since metastatic PCa and drug resistance are great challenges for clinical therapy, our findings shed light on understanding how localized PCa acquires necessary traits to become metastatic PCa and develop drug resistance. These findings suggest that miR-101, miR-27a, and COUP-TFII are potential novel targets for PCa therapy. Future advancement in specific delivery of miRNAs to PCa patients and identification of small molecules that inhibit COUP-TFII function will enhance the potential of development of more efficient cancer therapy for PCa.

## Methods

### Cell culture and treatment

LNCaP, PC3, C4-2 and 22RV-1 PCa cells were purchase from ATCC and maintained in Tissue and Cell Culture Core Facility at Baylor College of Medicine. Cells were cultured in RPMI1640 medium with 10% FBS and antibiotics (100 μg ml^−1^ streptomycin and 100 U ml^−1^ penicillin G) in a humidified atmosphere of 5% CO_2_ and 95% air at 37 °C. LNCaP-abl cells were cultured in RPMI1640 medium with 10% charcoal-stripped FBS. Fresh medium were changed after three days of incubation. Cells were routinely checked for mycoplasma contamination by using MycoAlert Mycoplasma Detection Kit (LONZA). Short-tandem repeat analysis was performed by the service of Core Facility in MD Anderson Cancer Center for the authentication of the cell lines. For the enzalutamide experiment, 2 × 10^3^ LNCaP parental and enzalutamide-resistant cells were plated into 96-well-plates and treated with 10 μM enzalutamide (purchased from Selleck Chemicals) for the indicated time points.

### siRNA and microRNA transfection

COUP-TFII, FOXM1 and CENPF siRNAs and miRNA mimics and inhibitors were purchased from Invitrogen. PCa cells were transiently transfected with 50 nM siRNA or miRNA reagents for 3 days using Lipofetamine 2000. To generate stable inhibition of endogenous miRNA function, lentiviral-based miRzip control vectors carrying GFP reporter genes and miRzip containing anti-miRNA sequences were purchased from System Biosciences. Virus was packaged in 293 T cells and PCa cell lines were infected with the virus for 2 days. Positive clones were then selected by puromycin selection.

### RT- qPCR

Total RNA was isolated by extraction with TRIzol reagent (Invitrogen) according to the protocol provided by the manufacturer. An amount of 100 or 500 ng total RNA was used for reverse transcription of miRNA and cDNA by TaqMan, MicroRNA Reverse Transcription Kit (Invitrogen) and MMLV reverse transcriptase (Promega), respectively. Subsequently, miRNA and mRNA transcripts were quantified by Applied Biosystems StepOnePlus real-time PCR (Invitrogen). Each reaction contained 40 ng miRNA-RT products, 1 × specific miRNA-PCR primer (Invitrogen) and 10 μl 2X Taqman reagent (Invitrogen) for miRNA detection. 50 ng cDNA products, 0.3 μM specific primer (Supplementary Table 4) and 10 μl SYBR Green mix (Roche) were used in each reaction for mRNA detection.

### Western blotting and immunohistochemical (IHC) staining

Total cell lysates were collected by RIPA buffer containing commercial protease inhibitors. 30 μg of protein was separated by SDS-polyacrylamide gel electrophoresis, and transferred to a polyvinylidene difluoride membrane. The membrane was blocked in 5% nonfat milk at room temperature for 1 h, followed by incubation with primary antibody prepared in 1 × PBST (PBS with 0.25% Tween 20) (COUP-TFII, 1:1000, PP-H7147-00, R&D systems; CENPF, 1:2000, Ab5, Abcam; FOXM1, 1:2000, #5436, Cell Signaling Technology; E-cadherin, 1:2000, #3195, Cell Signaling Technology; Vimentin, 1:2000, #5741 Cell Signaling Technology; GR, 1:2000, #12041, Cell Signaling Technology and AR, 1:2000, sc-816, Santa Cruz Biotechnology Inc.) at 4 °C overnight. Signals were developed using an enhanced chemiluminescence detection kit (PerkinElmer). For IHC staining, AR (1:1,000) antibody was purchased from Santa Cruz Biotechnology Inc (sc-816) and GFP (1:1,000) antibody was purchased from ThermoFisher (A11122). Uncropped scans of blots are shown in Supplementary Figs 15–17.

### Construction of an inducible plasmids and cell line

To set up inducible miR-101, miR-27a and COUP-TFII constructs, miRNA expression vectors were purchased (Origene Technologies, Inc) and construction of a COUP-TFII expression vector was described previously^13^. miR-101, miR-27a and COUP-TFII cDNAs were then amplified by primers (Supplementary Table 4) and cloned into pLVX-tight-Puro vector (Clontech Laboratories, Inc.). To set up the inducible stable clones, PC3 cells were infected with virus carrying rtTA constructs and selected by G418. PC3 Cells with rtTA were then infected with virus carrying pLVX-tight-miR-101, -miR-27a or -COUP-TFII before selection by puromycin. To generate inducible knockdown of COUP-TFII cells, shRNA against COUP-TFII was put into pLKO-Tet-on vector. LNCaP cells were infected with virus carrying inducible knockdown of COUP-TFII construct and stable clones were selected by neomycin.

### Reporter assays

A detailed procedure for cloning WT human COUP-TFII 3′-UTR into a PIS2 vector was illustrated previously^45^. Mutation of miR-101 and miR-27a recognition sites were generated by site-directed mutagenesis. The mutated primers were designed by QuikChange Primer Design Program provided by Agilent Technologies and listed in the Supplementary Table 4. To perform reporter assay, LNCaP and PC3 cells were transiently transfected with miRNA mimics, WT or mutated COUP-TFII-3'UTR constructs and β-gal as an internal control. After incubation for 48 h, luciferase and β-gal activities were measured.

### Cell invasion assays

Invasion chamber was prepared according to the datasheet (R&D Systems). A total of 5 × 10^4^ PCa cells were plated into the invasion chamber containing culture medium without FBS. Culture medium with 10% FBS was then added to the well and incubated for 16 h. After incubation, cells invaded to the bottom chamber were fixed with 4% paraformaldehyde and cells in the top chamber were removed. Invaded cells were stained by ReadyProbes reagent (Life Technologies Corporation) and counted from pictures taken at 9–11 different areas.

### Ago2-RNA-immunoprecipitation assay

Ago2-RIP experiment was performed by purchasing Ago2 antibody and RIP-assay kit from MBL International Corporation. The detailed procedures were as described in the datasheet. Protein and total RNA were extracted and assayed by western blot and RT-qPCR, respectively. The primers were individually designed to contain miR-101 or miR-27a recognition sites located in the COUP-TFII 3′-UTR region.

### Chromatin-immunoprecipitation assays

The ChIP assays were performed using Pierce^Tm^ Magnetic ChIP kit (Thermo Scientific). The procedure was as described in the kit provided by the manufacturer. Briefly, PC3 cells were fixed by 1% formaldehyde, fragmented by a combination of MNase and sonication. COUP-TFII antibody (R&D Systems) was then used for immunoprecipitation of DNA-COUP-TFII complexes. After washing and reverse-crosslinking, the precipitated DNA was amplified by primers and quantified by the StepOnePlus real-time-PCR machine. Primer sequences can be found in the Supplementary Table 4

### Animal models

All experiments were approved by the Animal Center for Comparative Medicine at Baylor College of Medicine. For the *ex vivo* metastasis model, 2 × 10^6^ PC3-Luciferase or 5 × 10^6^ LNCaP-Luciferase cells were injected into 6-week-old NOD-severe combined immunodeficient male mice via orthotopic injection. When tumour size reached 50 mm^3^, mice were randomly grouped and doxycycline (1 μg ml^−1^) was added into drinking water (For LNCaP cells). After incubation for 4 weeks (PC3) or 6 weeks (LNCaP), bioluminescence of LNCaP-Luciferase was measured by the *in vivo* imaging systems (IVIS).

### Expression profile and correlation as well as GSEA analysis

Two miRNA microarrays (GSE21036 and GSE26964) containing primary and metastatic PCa specimens were downloaded from GEO data sets and analysed by the GenePattern program. FDR <0.05 and fold change >2-fold were used as criteria to find the candidate miRNAs changed in metastatic PCa. Next, signatures for miR-101 and miR-27a were derived from GSE13674 and GSE65874 (genes with *P*<0.05, fold change >1.5-fold), respectively and further analysed by GSEA in a metastatic phenotype data set (GSE32269). For the correlation analysis, the levels of candidate miRNAs and genes were presented in a heatmap format.

### Signature analysis in prostate cancer patients

Gene transcription signature analysis of COUP-TFII, FOXM1, CENPF or miR-101 was based on previously described t-score metric^13^,^46^,^47^. COUP-TFII signature was derived from our previous data set GSE33182. FOXM1, CENPF and FOXM1-CENPF co-regulated signatures were downloaded from the Supplementary Table in Aytes^19^. miR-101 and miR-27a signatures (genes with *P*<0.01, fold change >1.5-fold) were derived from GSE13674 and GSE65874, respectively. PCa patient data sets were obtained from GSE21034 (Taylor), GSE10645 (Nakagawa) and TCGA PCa data sets. All the detail data set information was organized in the Supplementary Table 5.

### Metastatic gene regulatory network

To construct the metastatic gene regulatory network, genes involved in the cancer metastasis were exported from the MetaCore (578 genes) database and crossed to the gene list specifically changed (fold change>1.5-fold) in the clinical data set of metastatic PCa compared with the primary PCa. (GSE32269). Specific genes involved in the clinical metastasis of PCa were shown based on above criteria. Then, these selected genes automatically formed the metastatic gene regulatory network according to the literature reference from the Metacore database (shown in grey colour line). The relationships between upstream miRNA, COUP-TFII, FOXM1, CENPF and EMT regulators were annotated in solid black colour based on our findings. Red lines indicate the suppressive effect while green lines indicate the active effect. A solid black line indicates direct regulation.

### Statistical analysis

All numerical data are expressed as mean±s.e.m. For RT-qPCR, migration, invasion and proliferation assays, a paired two-tailed Student's *t*-test was used to compare differences between two groups and one-way AVNOVA followed by Dunnett post-analysis was used to compare differences more than two groups. Gene expression and signature correlations were performed by Pearson's correlation analysis. All the statistical analysis was performed by using commercial statistical software (GraphPad Prism 5.01, GraphPad Software). For all analyses, statistical significance was set at *P*<0.05.

### Data availability

Data referenced in this study are available in the GEO under the accession codes GSE21036, GSE26964, GSE13674, GSE65874, GSE32269, GSE33182, GSE21034 and GSE10645.

## Additional information

**How to cite this article**: Lin, S.-C. *et al*. Dysregulation of miRNAs-COUP-TFII-FOXM1-CENPF axis contributes to the metastasis of prostate cancer. *Nat. Commun.* 7:11418 doi: 10.1038/ncomms11418 (2016).

## Supplementary Material

Supplementary Information Supplementary Figures 1-17 and Supplementary Tables 1-5
