# Supplementary material for: Dysregulation of miRNAs-COUP-TFII-FOXM1-CENPF axis contributes to the metastasis of prostate cancer
Source: Nat Commun. 2016 Apr 25;7:11418. doi: 10.1038/ncomms11418 (PMC4848536; doi:10.1038/ncomms11418)
Supplement: Supplementary Information — Supplementary Figures 1-17 and Supplementary Tables 1-5 [file ncomms11418-s1.pdf]

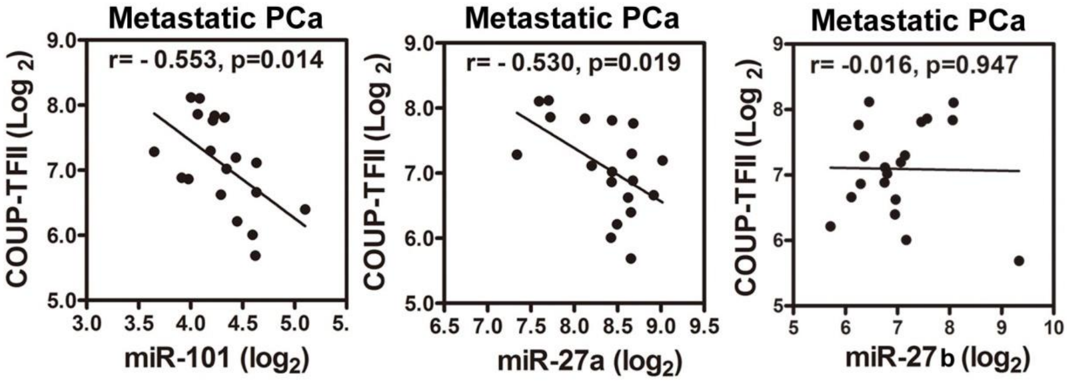

Supplementary Figure 1: Expression of COUP-TFII is negatively correlated with miR101 and miR27a but not with miR27b in metastatic prostate cancer specimens. Correlation analysis between the levels of COUP-TFII and miR-27b in metastatic prostate cancer (n=19). Pearson's correlation test was used.

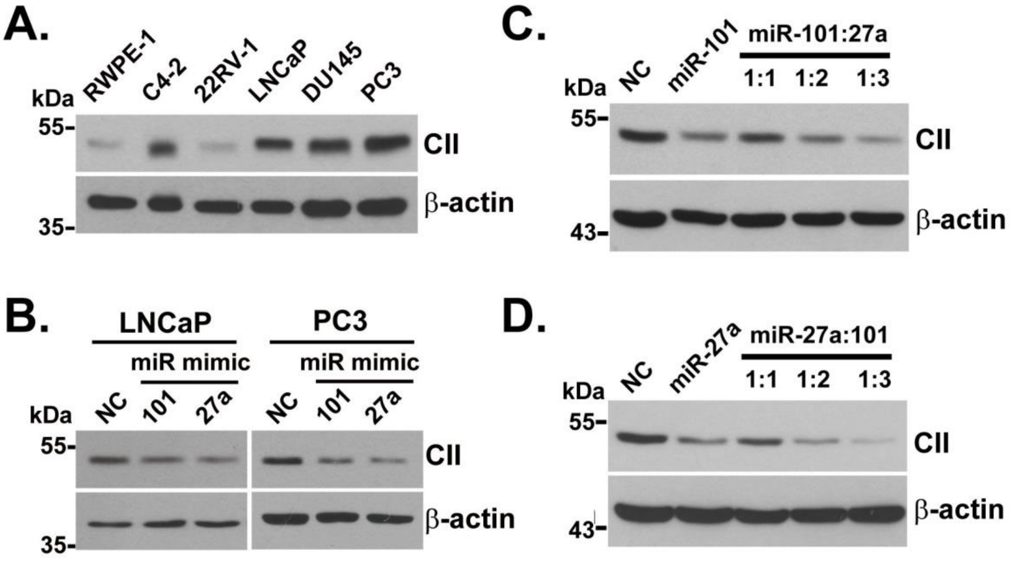

Supplementary Figure 2: Overexpression of miR-101 and miR-27a inhibited COUP-TFII expression. A. COUP-TFII in different prostate cancer cell line **B.** Representative Western blot showed the levels of COUP-TFII in LNCaP and PC3 cells overexpressed miR-101 and miR-27a for 72hrs. **C.** PC3 cells were individually treated with total amount of 40nM scramble control (NC), miR-101 , or several ratios of miR-101 versus miR-27a (1 unit equal to 10 nM and scramble control was used to adjust equal amount) for 72 hours. Then, COUP-TFII expression was measured by Western blot. A representative Western blot shows the expression of COUP-TFII in miRNA-overexpressed PC3 cells. **D.** The same experiment was performed by using overexpression of miR-27a alone and miR-27a versus miR-101 in PC3 cells

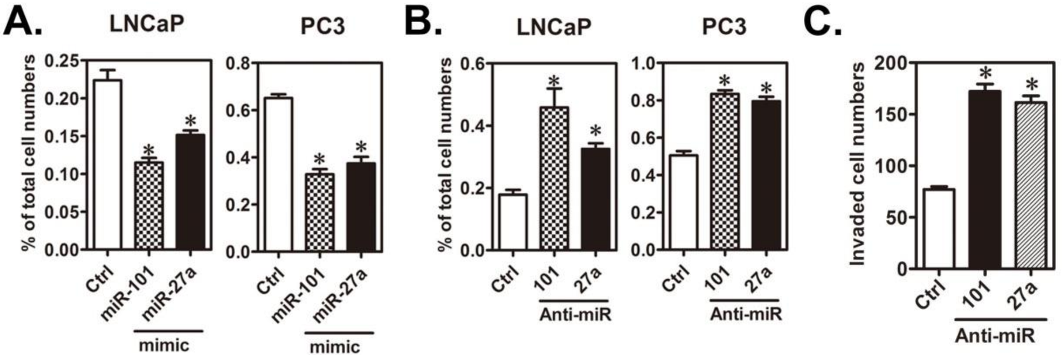

Supplementary Figure 3: miR-101 and miR-27a negatively regulate migration ability of prostate cancer cells. A, B. LNCaP and PC3 cells were treated with 50 nM mimic or anti-miR inhibitor for 72 hrs. Cells were trypsinized and performed migration chamber assay for 16 hrs (LNCaP) or 4 hrs (PC3) (n=3) *P<0.0001 (One-way ANOVA followed by Dunnett test). **C.** Stable clones of control, anti-miR-101 and anti-miR-27a in 22RV-1. Cells were used to perform the invasion assay (n=3). *P<0.0001 (One-way ANOVA followed by Dunnett test).

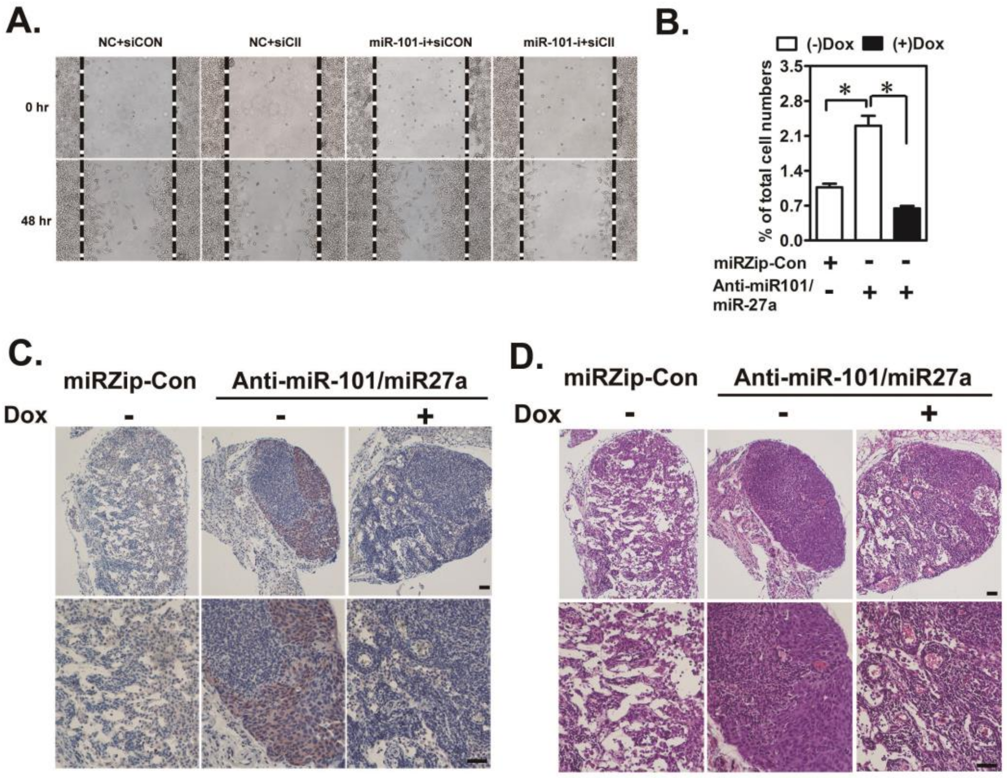

Supplementary Figure 4: Inhibition upstream miRNA-induced cell migration, invasion and metastasis were abrogated by knockdown of COUP-TFII expression. A. 22RV-1 cells were treated with miR-101 inhibitor and siRNA against COUP-TFII for 48hrs. Cells were performed scraped assay for additional 48hr. Representative picture showing the migrating cells in different experimental

conditions. **B.** Inducible knockdown of COUP-TFII LNCaP cells carrying with anti-miR-101 and anti-miR-27a constructs were treated with or without 1 mg/ml doxcycline for 48hrs. Cells were perfomed invasion assay for 16hrs (n=3). *P<0.05 (two-sided Student's t-test) **C.** Representative GFP staining in the mouse lymph node isolated from different groups. **D.** Representative hematoxylin and eosin stain (H&E stain) in the mouse lymph node isolated from different groups.

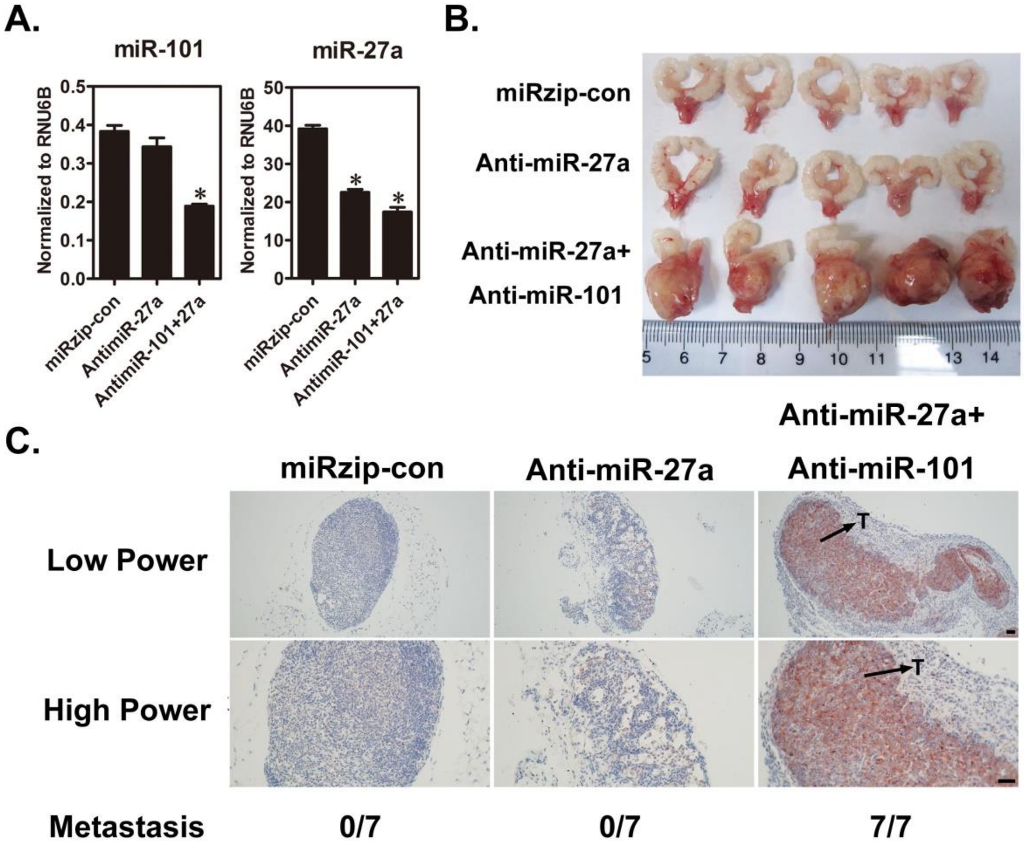

Supplementary Figure 5: Inhibition of miR-101 and miR-27a promotes tumor growth and lymphatic metastasis in orthotopic injection model of prostate cancer. **A.** PC3 cells were infected with con, anti-miR-27a or anti-miR-101 and anti-miR-27a viruses to set up the stable clones. RT-qPCR results showed the anti-miR construct inhibited miR-101 and miR-27a expression (n=3). *P<0.05 (two-sided Student's t-test). **B.** Representative picture of prostate tumor isolated from orthotopic injection mouse model of prostate cancer after inoculation for 4 weeks. **C.** Representative GFP staining in the mouse lymph node isolated from different groups.

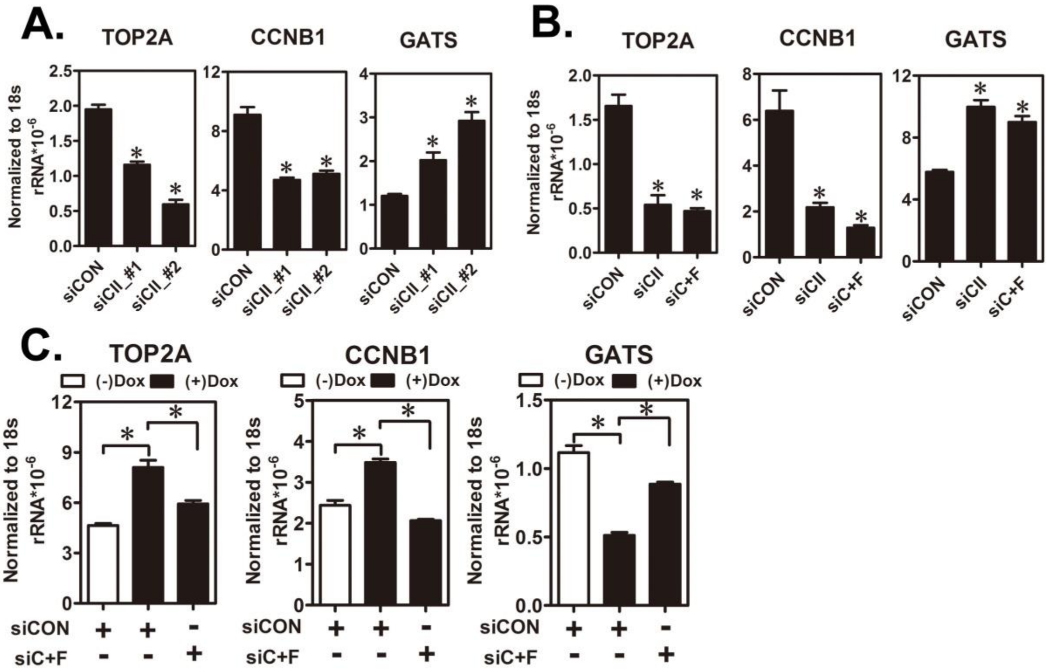

Supplementary Figure 6: Regulation of CENPF and FOXM1 downstream target gene expression by COUP-TFII. A. RT-real-time PCR data demonstrate that CENPF and FOXM1 downstream target genes, TOP2A and CCNB1 were downregulated while GATS was increased after knockdown of COUP-TFII (siCII) for 72 hours in PC3 cells (n=3). *P<0.001 (One-way ANOVA followed by Dunnett test). **B.** Similar results were also observed in LNCaP cells after knockdown of COUP-TFII or double-knockdown of CENPF and FOXM1 (siC+F) for 72 hours (n=3). *P<0.001 (One-way ANOVA followed by Dunnett test). **C.** PC3 cells harbor an inducible COUP-TFII construct were knocked down with both CENPF and FOXM1 siRNAs (siC+F) and treated with water or 1 ug/ml doxycycline for 72 hours to induce COUP-TFII expression (n=3). *P<0.05 (two-sided Student's t-test).

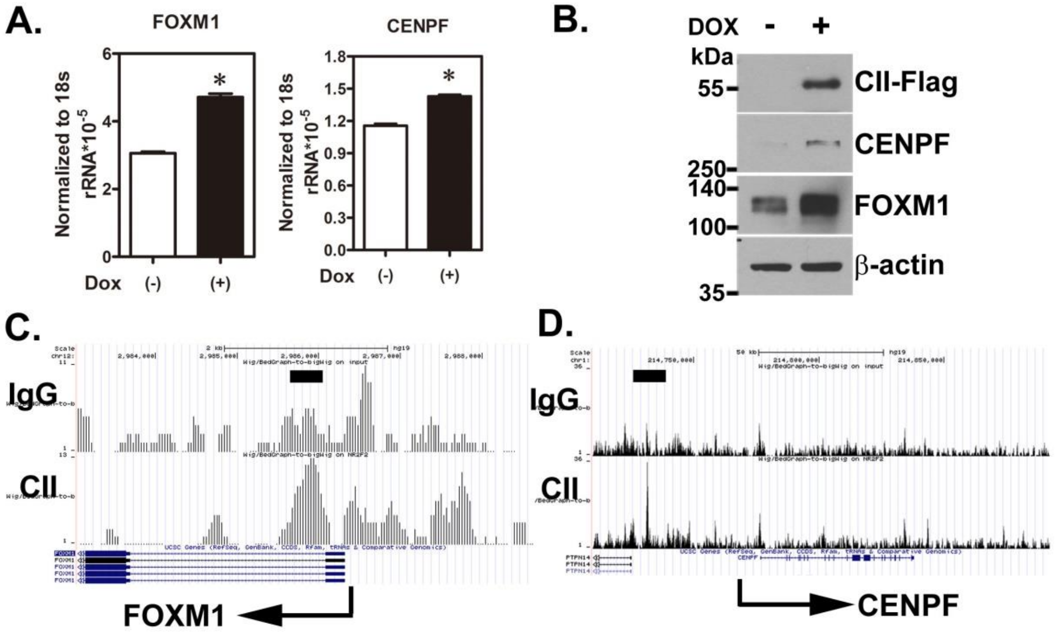

Supplementary Figure 7: COUP-TFII directly increased FOXM1 and CENPF expressions. A. PC3 cells carrying with inducible COUP-TFII-Flag were treated with 0.2 mg/ml doxycycline for 72hrs. Levels of FOXM1 and CENPF were measured by RT-qPCR (n=3). *P<0.05 (two-sided Student's t-test) **B.** Representative Western blot showing the levels of COUP-TFII-Flag, FOXM1, CENPF and b-actin. **C, D** COUP-TFII binding profile in FOXM1 and CENPF locus. COUP-TFII ChIP-seq was analyzed from GSE52008. Black rectangle marks the peak calling region for COUP-TFII .

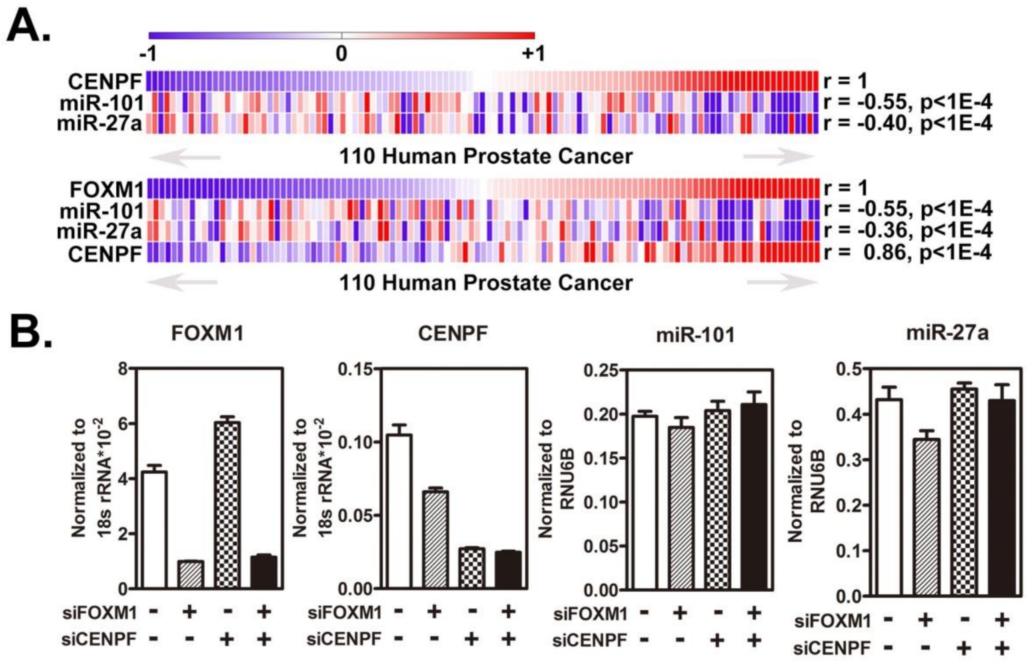

Supplementary Figure 8: Both CENPF and FOXM1 expression are negatively correlated with miRNA-101 and miRNA-27a expression. A. Heatmap showed the negative correlation between the levels of CENPF, miR-101 and miR-27a (upper panel) and FOXM, CENPF, and those miRNAs (lower panel) in Taylor dataset (GSE21034 and GSE21036). Pearson's correlation test was used. **B.** FOXM1 and CENPF failed to reglate miR-101 and miR-27a expression**.** LNCaP cells were individually or concomitantly treated with siRNA against FOXM1 or CENPF for 72hrs. Levels of FOXM1, CENPF, miR-101and miR-27a were measured by RT-qPCR (n=3).

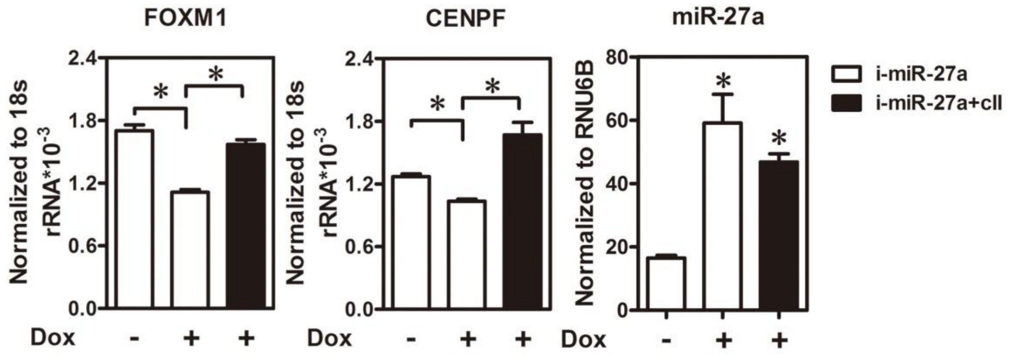

Supplementary Figure 9: Repression of FOXM1 and CENPF by miR-27a was rescued by ectopic overexpression of COUP-TFII. PC3 cells carrying with inducible miR-27a or inducible miR-27a overexpressed ectopic COUP-TFII were treated with 0.2 mg/ml doxycycline for 96hrs. Levels of FOXM1, CENPF and miR-27a were measured by RT-qPCR (n=3). *P<0.05 (two-sided Student's t-test).

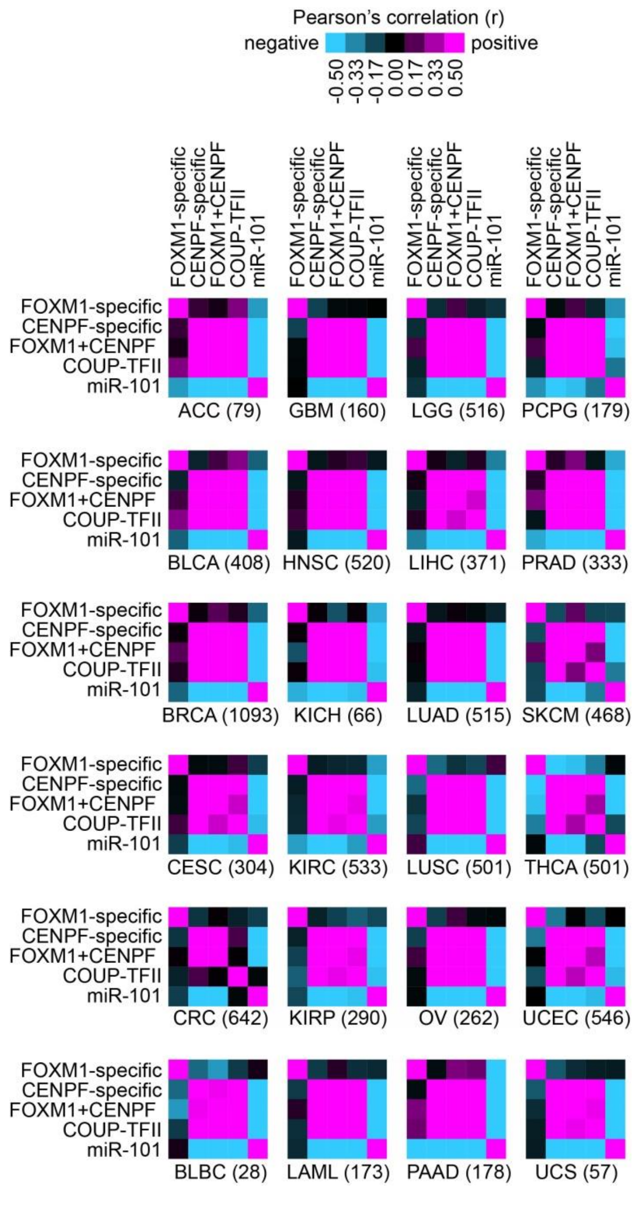

Supplementary Figure 10: Regulation cascade of miR-101-COUP-TFII-CENPF-FOXM1 is a general phenomeon in different cancer types. Pan-cancer analysis, examining correlations between five different gene signatures (FOXM1-specific, CENPF-specific, FOXM1+CENPF, COUP-TFII, miR-101), for each of 24 different cancer types represented in The Cancer Genome Atlas (TCGA). For each cancer type, five different gene signature scores (FOXM1-specific, CENPF-specific, FOXM1+CENPF, COUP-TFII, miR-101) were generated for each sample profile, and the scores for the various signatures were correlated between each other across the samples. Numbers of tumors used in the analysis for each cancer type are indicated, next to the corresponding TCGA project abbreviation.

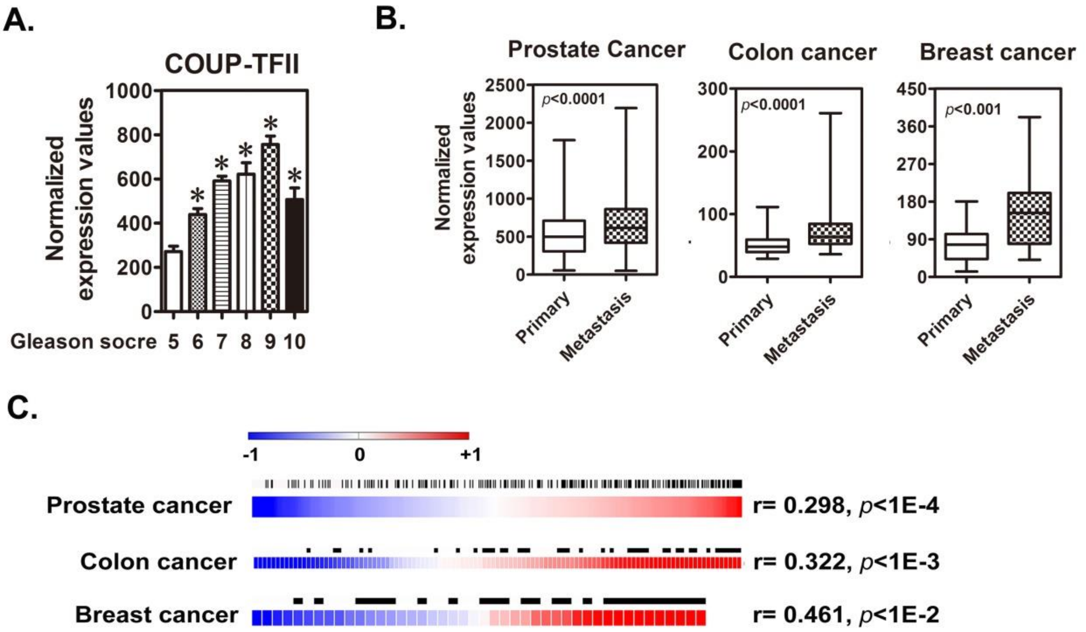

Supplementary Figure 11: COUP-TFII expression is an indicator for prediction of metastatic prostate cancer. **A.** The level of COUP-TFII expression was analyzed according to Gleason score of prostate cancer. *P<0.0001 (One-way ANOVA followed by Dunnett test). **B.** COUP-TFII expression in prostate (GSE46691; Primary (n=332), Metastasis (n=213)), colon (GSE27854; Primary (n=74), Metastasis (n=40)) and breast cancer (GSE56493; Primary (n=18), Metastasis (n=24)). *P<0.05 (two-sided Student’s t-test) **C.** Pearson’s correlation analysis between COUP-TFII expression and metastatic event.

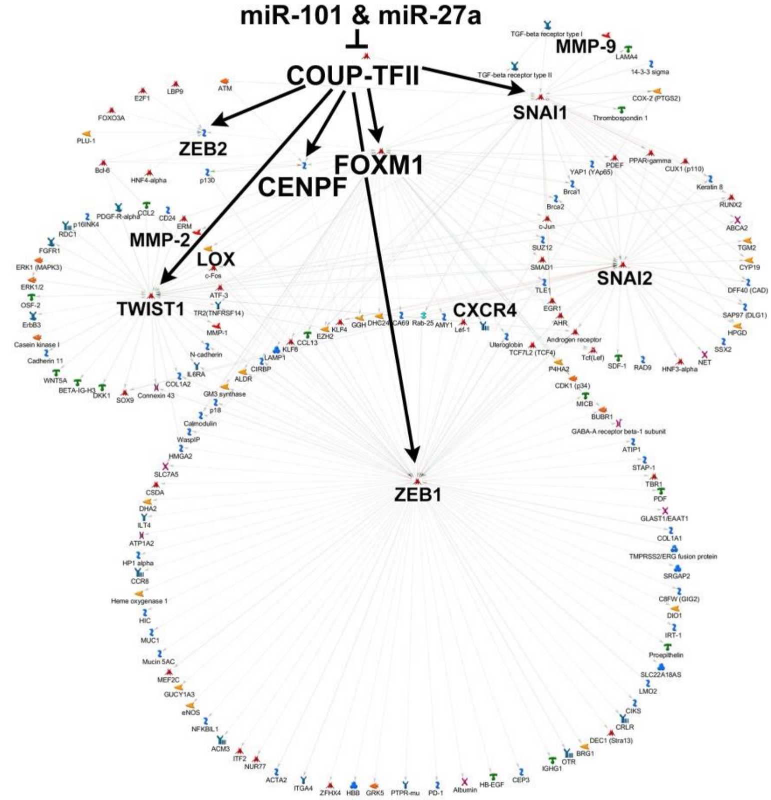

Supplementary Figure 12: Metastatic gene regulatory network in prostate cancer. The network was constructed by MetaCore. Red lines indicate the suppressive effect while green lines indicate the active effect. Solid black line indicates the direct regulation.

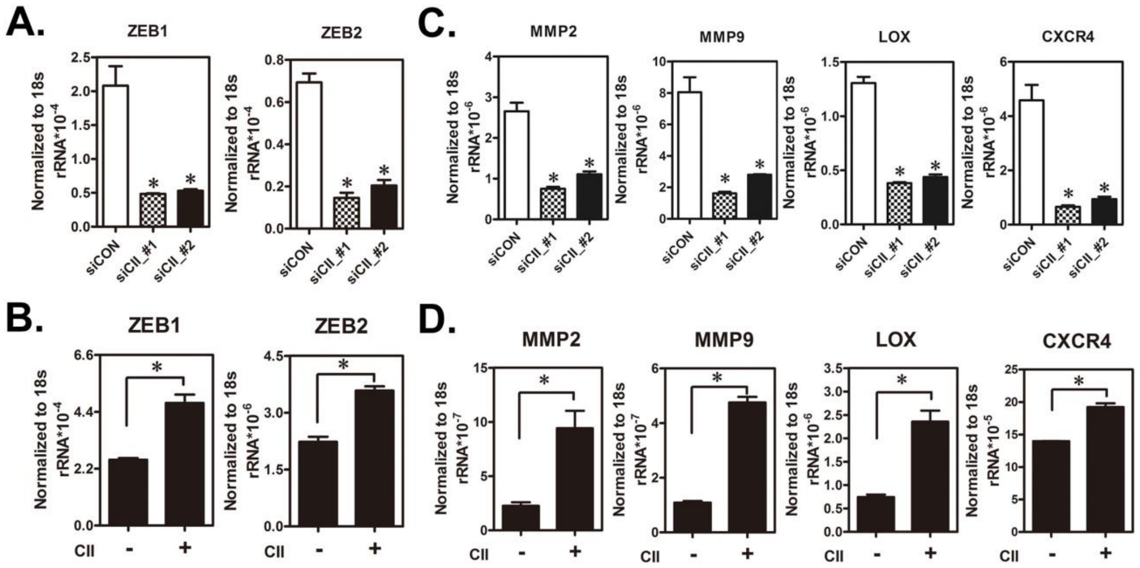

Supplementary Figure 13: miRNA regulation of MMP2, MMP9, LOX and CXCR4 expression is through downregulation of COUP-TFII. A, C. PC3 cells were treated with two different siRNAs against COUP-TFII (20 nM) for 72hrs. Levels of MMP2, MMP9, LOX and CXCR4 were determined by RT-qPCR (n=3). *P<0.0001 (One-way ANOVA followed by Dunnett test). **B, D.** PC3 cells carrying

with inducible COUP-TFII-Flag were treated with or without 0.2 mg/ml doxycycline for 72hrs. Levels of MMP2, MMP9, LOX and CXCR4 were determined by RT-qPCR (n=3). *P<0.05 (two-sided Student's t-test)

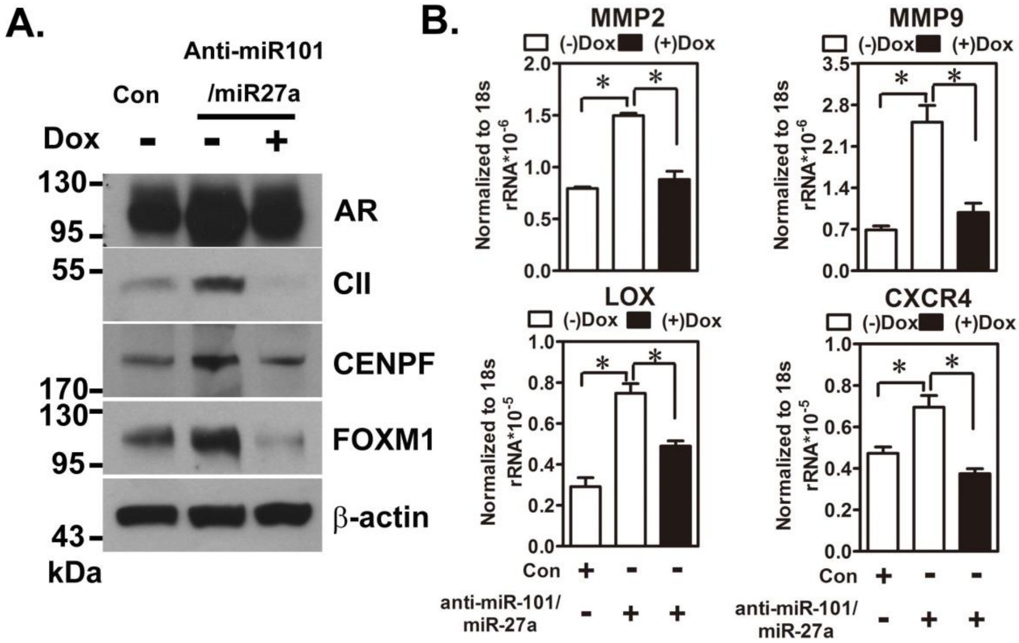

Supplementary Figure 14: Inhibition upstream miRNA-increased metastasis-related gene expression was mediated through COUP-TFII upregulation . Inducible knockdown of COUP-TFII LNCaP cells carrying with anti-miR-101 and anti-miR-27a constructs were treated with or without 1 mg/ml doxcycline for 72hrs **A**. Representative Western blot figure for COUP-TFII, CENPF and FOXM1 expressions. **B.** Levels of MMP2, MMP9, LOX and CXCR4 were determined by RT-qPCR (n=3). *P<0.05 (two-sided Student's t-test) .

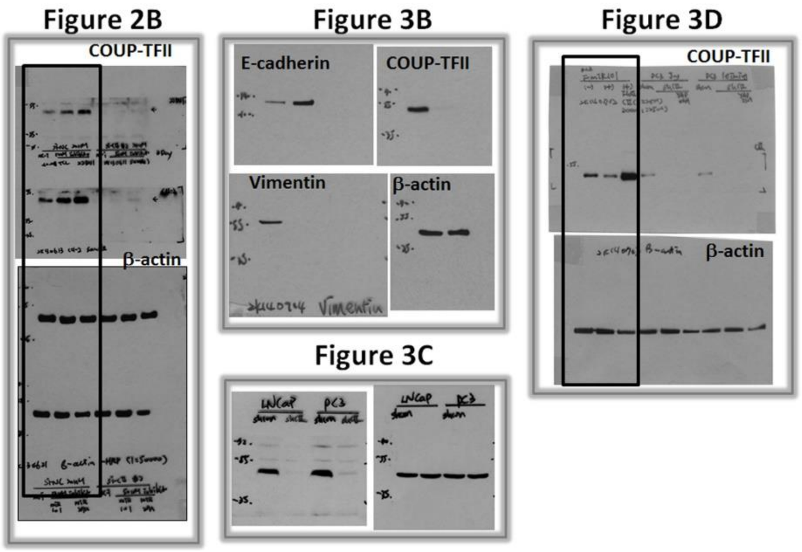

Supplementary Figure 15: Full scan images of Figure 2B , 3B, 3C and 3D

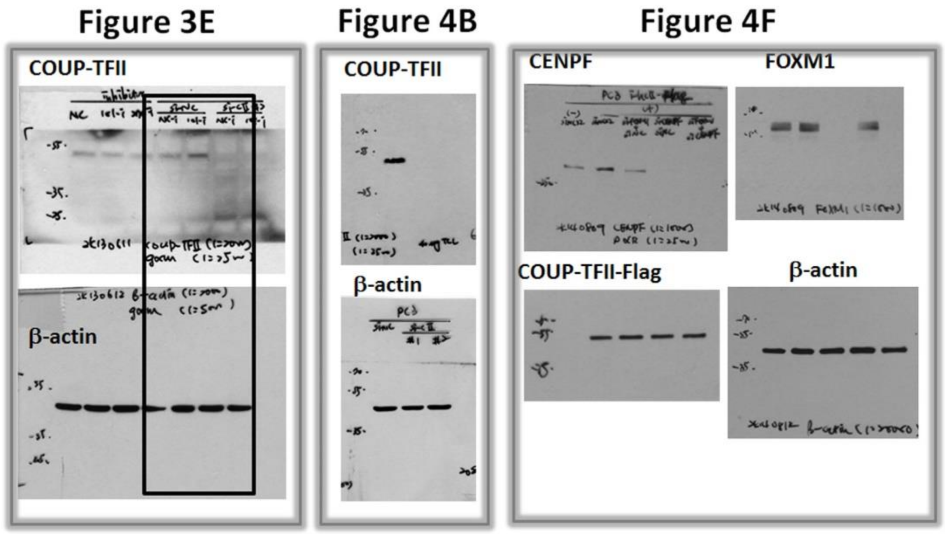

Supplementary Figure 16: Full scan images of Figure 3E, 4B and 4F

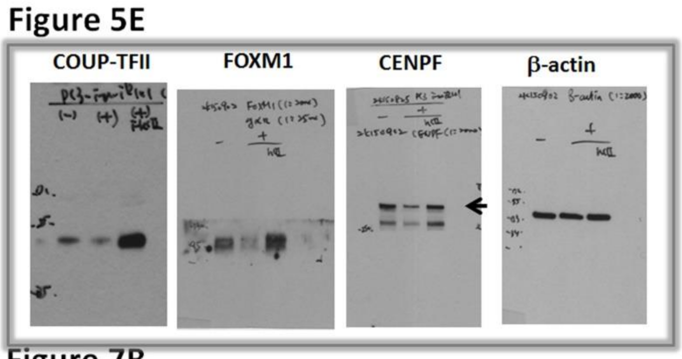

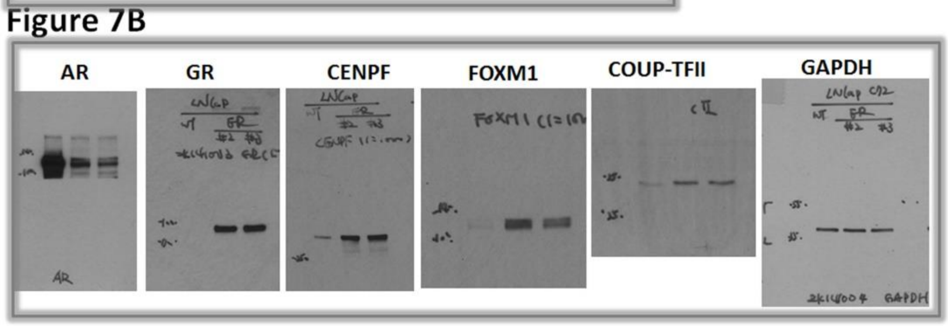

Supplementary Figure 17: Full scan images of Figure 5E and 7B

Supplementary Table 1: miRNAs changed in metastatic Pca					
**FDR<0.05, Fold change > 2 Fold**					
**31**	**GSE21034**	**GSE26964**	**2**	**GSE21034**	**GSE26964**
**miR_down regulation**	**log2**	**log2**	**miR_Up regulation**	**log2**	**log2**
**hsa-let-7e**	**-1.18**	**-6.96**	**hsa-miR-371-5p**	**1.01**	**7.65**
**hsa-miR-1**	**-5.62**	**-32.62**	**hsa-miR-575**	**1.35**	**3.80**
**hsa-miR-100**	**-2.13**	**-6.81**			
**hsa-miR-101**	**-1.10**	**-8.63**			
**hsa-miR-125b**	**-1.86**	**-5.91**			
**hsa-miR-130a**	**-2.13**	**-12.20**			
**hsa-miR-135a**	**-2.35**	**-5.71**			
**hsa-miR-136**	**-1.20**	**-14.98**			
**hsa-miR-143**	**-4.50**	**-29.15**			
**hsa-miR-145**	**-3.84**	**-26.22**			
**hsa-miR-152**	**-1.27**	**-15.33**			
**hsa-miR-199b-5p**	**-2.36**	**-56.10**			
**hsa-miR-204**	**-2.23**	**-12.74**			
**hsa-miR-205**	**-6.99**	**-134.13**			
**hsa-miR-218**	**-1.81**	**-30.10**			
**hsa-miR-221**	**-2.78**	**-84.88**			
**hsa-miR-222**	**-2.34**	**-57.64**			
**hsa-miR-224**	**-1.07**	**-1.38**			
**hsa-miR-23a**	**-1.06**	**-8.73**			
**hsa-miR-23b**	**-2.03**	**-8.51**			
**hsa-miR-24**	**-1.16**	**-7.01**			
**hsa-miR-27a**	**-1.06**	**-5.48**			
**hsa-miR-27b**	**-1.81**	**-6.85**			
**hsa-miR-30c**	**-1.10**	**-6.44**			
**hsa-miR-374a**	**-1.41**	**-10.10**			
**hsa-miR-376a**	**-1.54**	**-10.18**			
**hsa-miR-376c**	**-1.56**	**-9.15**			
**hsa-miR-377**	**-1.48**	**-12.03**			
**hsa-miR-455-3p**	**-1.42**	**-1.44**			
**hsa-miR-455-5p**	**-1.12**	**-3.07**			
**hsa-miR-495**	**-1.03**	**-9.13**			
**hsa-miR-582-5p**	**-1.46**	**-1.38**			

Supplementary Table 1: miRNA changed in metastatic PCa

Supplementary Table 2: Signature analysis in Nakagawa prostate cancer dataset.

Nakagawa Dataset	miR-101 signature	miR-27a signature
**NR2F2 signature**		
Correlation coefficient	-0.655	-0.481
Valid cases	596	596
One-sided significance	1.408E-74	4.221E-36
**FOXM1-specific**		
Correlation coefficient	-0.180	-0.012
Valid cases	596	596
One-sided significance	4.739E-06	3.883E-01
**CENPF-specific**		
Correlation coefficient	-0.386	-0.379
Valid cases	596	596
One-sided significance	6.210E-23	4.339E-22
**FOXM1+siCENPF**		
Correlation coefficient	-0.205	0.097
Valid cases	596	596
One-sided significance	2.327E-07	8.652E-03

Supplementary Table 2: Signature analysis in Nakagawa prostate cancer dataset

Supplementary Table 3: Multivariate Cox analysis

Multivariate Cox	*P* value	HR	95% CI
COUP-TFII signature	0.0002	1.25	1.11-1.40
miR-101 signature	0.01	0.86	0.76-0.96
miR-27a signature	<0.0001	0.7	0.61-0.80

Supplementary Table 3: Multivariate Cox analysis

Supplementary Table 4: Primer List	
Primer	Sequence (5'-->3')
mutation-miR-101 Sense	aggtttttttttttaagtgcaacatttctgtatgatggaaaagttataataactgaactgtttggtcgag
mutation-miR-101 Antisense	ctcgaccaaacagttcagttattataacttttccatcatacagaaatgttgcacttaaaaaaaaaaacct
mutation-miR-27a Sense	catttgacagtctttttttttttttttgaaatacattgtccattaaattcttcccctctcttctttta
mutation-miR-27a Antisense	taaaagaagagaggggaagaatttaatggacaatgtatttcaaaaaaaaaaaaaaagactgtcaaatg
ZEB1 realtime primer F	gtggcggtagatggtaatgt
ZEB1 realtime primer R	cttgttgtatgggtgaagca
ZEB2 realtime primer F	tttcctgccctctctgtagc
ZEB2 realtime primer R	ccttgggttagcatttggtg
MMP2 realtime primer F	gtatggcttctgccctgaga
MMP2 realtime primer R	cacaccacatctttccgtca
MMP9 realtime primer F	gaaccaatctcaccgacagg
MMP9 realtime primer R	cgactctccacgcatctct
LOX realtime primer F	ttacccagccgaccaagata
LOX realtime primer R	ccttcagccactctcctctg
CXCR4 realtime primer F	ttcctgcccaccatctactc
CXCR4 realtime primer R	tccgtcatgcttctcagtttc
FOXM1 realtime primer F	ttctcctttgcttccagttca
FOXM1 realtime primer R	cactttgatgggtctcgctaa
CENPF realtime primer F	aaagaaacagacggaacaactg
CENPF realtime primer R	ccaagcaaagaccgagaact
TOP2A realtime primer F	AAACTCGATGATGCCAATGA
TOP2A realtime primer R	GTCTCTCCCAACCACACCAA
CCNB1 realtime primer F	CTTGCAGTAAATGATGTGGATG
CCNB1 realtime primer R	GTGACTTCCCGACCCAGTAG
GATS realtime primer F	AGAGTATCCCGCTGTTCACC
GATS realtime primer R	GAATCCTTCCTCATCGACAA
RNA-IP realtime primer for miR-101 F	ggcacttataggtaacgtgatt
RNA-IP realtime primer for miR-101 R	acacaaagactcgaccaaac
RNA-IP realtime primer for miR-27a F	ggtttgtttgcttaatttcct
RNA-IP realtime primer for miR1-27a R	tcacagtccattaaattcttcc
CIIBS-ChIP-realtime primer for FOXM1-	cgactgtggctgagatgaag
CIIBS-ChIP-realtime primer for FOXM1-	gtaagatggaggcggtgttg
CIIBS-ChIP-realtime primer for CENPF-	GTGTTTGGGATGCCTTTCT
CIIBS-ChIP-realtime primer for CENPF-	TTACACAACCCTGCCCATA
CIIBS-ChIP#1-realtime primer for ZEB1-	ggctgagacataatccacatc
CIIBS-ChIP#1-realtime primer for ZEB1-	attgccttgagattgcttgt
CIIBS-ChIP#2-realtime primer for ZEB1-	acaagctaatcccacccata
CIIBS-ChIP#2-realtime primer for ZEB1-	aggcactatacaatctggcact
CIIBS-ChIP-realtime primer for ZEB2-F	agtcccacgaaattaaaccac
CIIBS-ChIP-realtime primer for ZEB2-R	gagacaagctgttgctacttca
siRNA for COUP-TFII#1_sense	GCUUUGGAAGAAUACGUUAtt
siRNA for COUP-TFII#1_Antisense	UAACGUAUUCUUCCAAAGCac
siRNA for COUP-TFII#2_sense	CCUCCUCAGUCAUAGAGCAtt
siRNA for COUP-TFII#2_Antisense	UGCUCUAUGACUGAGGAGGag

Supplementary Table 4: Primer List

Supplementary Table 5: information of dataset		
**Data Source**	**Dataset information**	**Reference**
**GSE20136**	**miRNA expression profile in primary and metastatic PCa specimens**	**1**
**GSE26964**	**miRNA expression profile in primary and metastatic PCa specimens**	**none**
**GSE13674**	**overexpresion of miR-101 in UM-UC-3**	**2**
**GSE65874**	**overexpresion of miR-27a in 70/3 mouse pre-B cells**	**none**
**GSE32269**	**gene expression profile in primary and metastatic PCa specimens**	**3**
**GSE33182**	**knockdown of COUP-TFII in PC3 cancer cells**	**4**
**GSE21034**	**gene expression profile in PCa specimens**	**1**
**GSE10645**	**gene expression profile in PCa specimens**	**5**
**GSE52008**	**COUP-TFII ChIP-seq**	**6**

Supplementary Table 5: Information of dataset

Supplementary References

1. Taylor, B.S., *et al.* Integrative genomic profiling of human prostate cancer. *Cancer cell* **18**, 11-22 (2010).
2. Friedman, J.M., *et al.* The putative tumor suppressor microRNA-101 modulates the cancer epigenome by repressing the polycomb group protein EZH2. *Cancer research* **69**, 2623-2629 (2009).
3. Cai, C., *et al.* ERG induces androgen receptor-mediated regulation of SOX9 in prostate cancer. *The Journal of clinical investigation* **123**, 1109-1122 (2013).
4. Qin, J., Tsai, S. & Tsai, M.J. COUP-TFII, a prognostic marker and therapeutic target for prostate cancer. *Asian journal of andrology* **15**, 360-361 (2013).
5. Nakagawa, T., *et al.* A tissue biomarker panel predicting systemic progression after PSA recurrence post-definitive prostate cancer therapy. *PloS one* **3**, e2318 (2008).
6. Li, X., *et al.* COUP-TFII regulates human endometrial stromal genes involved in inflammation. *Molecular endocrinology* **27**, 2041-2054 (2013).
